# ALEE-AMC: a standardizable method to determine evolutionary adaptation of bacteria on non-porous surfaces with antibacterial activity

**DOI:** 10.1038/s44259-026-00297-2

**Published:** 2026-11-12

**Authors:** Niclas Nordholt, Selina B. I. Schmidt, Viola Boenke, Claudia Sündermann, Susann Wiltner, Beatrice Anger, Charlotte Valentin, Arnaud Bridier, Christophe Soumet, Alexander J. Cunliffe, James Redfern, Gillian Iredale, Peter D. Askew, Diana C. Mogrovejo, Marina Ruhlandt, Jan-Hendrik Klock, Florian H. H. Brill, Merilin Rosenberg, Angela Ivask, Frank Schreiber

**Affiliations:** 1 Department of Materials and the Environment, Division of Biodeterioration of Materials (4.1), Federal Institute for Materials Research and Testing (BAM), Berlin, Germany; 2 Antibiotics, Biocides, Residues and Resistance unit, French Agency for Food, Environmental and Occupational Health & Safety (Anses), Fougères Laboratory, Bâtiment Bioagropolis, Javené, France; 3 Department of Natural Sciences, Faculty of Science and Engineering, Manchester Metropolitan University, Manchester, UK; 4 Industrial Microbiological Services Limited (IMSL), Hants, UK; 5 Microbiology Department, Dr. Brill + Partner GmbH Institute for Hygiene and Microbiology, Hamburg, Germany; 6 Institute of Molecular and Cell Biology, University of Tartu, Tartu, Estonia; 7 Present Address: Department of Biochemistry, Charité – Universitätsmedizin Berlin, Berlin, Germany; 8 Present Address: SLB, Bergen, Norway

**Keywords:** Biotechnology, Microbiology

## Abstract

Evolutionary adaptation of bacteria to antimicrobial materials and coatings (AMCs) threatens their efficacy and may lead to cross-resistance to antibiotics. We develop an adaptive laboratory evolution experiment to be performed on AMCs (ALEE-AMC) based on the efficacy standard ISO 22196:2011, using metallic copper as a reference AMC and *Escherichia coli* as a model microorganism. The method consists of three steps: (i) determination of a timepoint at which viable cells are reduced upon AMC exposure by ~1000-fold, (ii) repeated cycles of exposure to AMCs and re-growth of surviving cells, and (iii) isolation of clones from the evolved populations and phenotypic testing. Repeated exposure to copper consistently selects for increased survival of evolved populations across participants of a ring trial. We find that laboratory identity did neither have a statistically significant effect on the average adapted antibacterial activity nor on the number of replicate populations that increased their survival to >100-fold. Antimicrobial susceptibility testing of isolated clones and whole genome sequencing showed that ALEE-AMC can detect changes in susceptibility including those above the clinical breakpoint related to mutations in known resistance determinants; specifically, cross-resistance to fosfomycin was observed in several copper-evolved clones. ALEE-AMC offers a standardizable platform to assess the risk of bacterial adaptation to AMCs.

## Introduction

Transfer of bacteria via surfaces contributes to the transmission of pathogens and is an important driver of infections acquired within sensitive settings such as hospitals, elderly care units, childcare, food industry or high-touch, open public places^1^. Antimicrobial materials, surfaces and coatings (AMCs) are designed and applied to reduce the burden of viable pathogenic bacteria on surfaces and thereby reduce the transmission of these bacteria between humans^2,3^. We use the term AMC in a broad sense, including non-antimicrobial materials coated with an antimicrobial active layer and materials that have intrinsic antimicrobial properties (e.g., metallic copper). However, the antimicrobial effect of AMCs may promote the risk of selection of adapted microorganisms via two main mechanisms: (i) acquisition of new mutations or genes via de novo evolution or horizontal gene transfer, respectively, linked to subsequent selective survival on the AMC and growth upon transfer to a new habitat, and (ii) ecological species sorting of inherently resistant strains upon exposure to the AMC^4^. These processes pose two risks: (i) AMCs may lose their efficacy over time and (ii) AMCs may promote the co-selection of bacterial strains that are resistant to other antimicrobial compounds, including strains with cross- or co-resistance to antibiotics and other antimicrobials such as disinfectants or antiseptics^5^.

The sale and use of AMCs are regulated in many countries. For example, the biological activity of AMCs classifies them as regulated according to the European Biocidal Product Regulation (BPR)^6^. Besides the assessment of toxicity to humans and the environment, the BPR requires the assessment of the efficacy of AMCs and the possible unacceptable occurrence of resistance. In this context, resistance is defined as a relevant reduction in susceptibility of the target organism(s) that renders the AMC ineffective^7^. Efficacy of an AMC that has the purpose of reducing the number of viable bacterial cells is measured by the survival of cells in contact with the AMC. The potential to evolve resistance to the AMC regarding this claim (reduction of viable cells) should thus be measured as the ability for evolutionary adaptation towards “increased survival”. In the field of antibiotics, the phenomenon of “increased survival” has been defined as tolerance^8^. Unfortunately, the term tolerance has not been used with a common definition in the field of antibiotics and AMCs^9,10^. We therefore refrain from using the term tolerance in this study, referring instead to the term “increased survival”, which is a relative quantity that can be readily determined. In order for “increased survival” to be categorized as resistance to an AMC that has a claim of reduction of viable cells, the reduction must be relevant. The conditions for efficacy are captured in regulatory guidelines. For example, current BPR guidelines require an efficacy of AMCs with desired disinfecting properties in medical settings to reach an antibacterial activity *R* = 3 (i.e., a 3 log or 1000-fold reduction of viable cells) within 60 min exposure time using standardized testing^11^. Regulatory assessment of products is strongly facilitated by standardized methods, allowing companies to test their products with widely accepted, well-described methods and providing comparable data. While a number of methods for the assessment of AMC efficacy have been described and standardized^12^, there is a lack of standardizable methods that support the assessment of the probability of evolutionary adaptation that renders the product ineffective due to “increased survival” (i.e., resistance to the AMC) and accounts for the potential of co-selection of antibiotic resistance.

ISO 22196:2011 is a widely used and relatively simple standard to test the antibacterial activity of non-porous surfaces under wet conditions^13^. In ISO 22196:2011, a thin, liquid film of a bacterial suspension (400 µL of ~6 × 10^5^ cells/mL on a 4 × 4 cm area) is held in contact with the AMC using a plastic film, incubated for 24 h followed by removal of the cells into a neutralizing solution, and plate counting viable cells after re-growth of colonies on agar. This method allows the demonstration of basic antibacterial activity of an AMC via leaching of active substances into the liquid cell suspension or by contact killing. The method is limited for the assessment of AMC efficacy by the difficulty in translating the results to end-use settings in which bacteria are transmitted to dry surfaces^12,14^. However, the approach taken by ISO 22196:2011 has the potential to serve as a basis to develop a standard to assess the *potential* for evolutionary adaptation because the method is standardized, widely used, easy to implement, and it allows a high level of experimental control and reproducibility in the treatment of cells across repeated exposure cycles^15^. A modification of the method for an adaptive laboratory evolution experiment (ALEE)^16^ is hampered by two test parameters: (i) ISO 22196:2011 requires a relatively low inoculum of cells (~6 × 10^5^ cells/mL) and (ii) a relatively long exposure time (24 h). Both parameters may result in killing all the inoculated bacteria, hampering detection of the potential for a selective advantage of de novo evolving mutants. In this study, both parameters were modified to enable repeated exposure cycles compatible with an ALEE.

Metallic copper (designated copper in the following for brevity if not stated otherwise) is among the best-established antimicrobial materials to date. The efficacy of copper against bacteria is well-established such that antibacterial claims for certain copper-based materials are approved by the American Environmental Protection Agency^17^. In addition, real-life intervention trials have been performed showing that copper surfaces can reduce the microbial burden under real-life conditions^18,19^ while its impact on reducing infections under these conditions is debated^20,21^. Several studies have investigated the mechanisms of metallic copper killing, showing the involvement of leached copper ions and contact killing in the process, as well as the underlying molecular mechanisms involved in the toxicity of copper for bacteria^22–28^. Three studies have already performed ALEEs with *Escherichia coli*, *Staphylococcus aureus* or *Pseudomonas fluorescens* on copper surfaces using different protocols^29–31^. All those studies were able to show that bacteria can adapt towards increased survival upon repeated exposure to copper, indicating that copper might be a suitable AMC serving as a reference for the development of a standardized test method to investigate evolutionary adaptation upon exposure to an AMC. However, none of the studies was able to associate the evolved phenotypes with a clear molecular mechanism, and the rate and magnitude of adaption were different between the studies. The main differences between the studies were the methods employed to inoculate the surfaces, the copper used, and the details on how to cycle bacteria between phases of killing and growth. Therefore, the observed differences might be related to different aspects of the underlying experimental protocols, showing that standardized procedures would be beneficial for an improved understanding and assessment of evolutionary adaptation to AMCs.

The aim of this study is to modify ISO 22196:2011, an internationally accepted standard method for efficacy testing of AMCs, in order to use the method to determine the potential of bacteria to adapt by evolving increased survival to AMCs due to repeated exposure by de novo mutation and selection of mutated clones. Copper was used as a model AMC and *E. coli* ATCC 8739 was used as a model microorganism because it is the strain required by ISO 22196:2011. First, we modified ISO 22196:2011 by (i) changing the pre-culture conditions to prepare the inoculum from a liquid culture instead of an agar-grown colony, (ii) increasing the cell density in the exposed cell suspension, and (iii) changing the exposure time to a duration that leads to a 3 log reduction of viable cells as required by current efficacy assessment guidelines. Next, we used the newly established method to investigate evolution for increased survival of *E. coli* upon repeated exposure to copper, establishing a reference procedure and a reference dataset. We then conducted a ring trial involving six additional participating laboratories. The outcomes of the interlaboratory test were compared according to different endpoints: (i) antibacterial activity of copper against evolved populations, (ii) antibacterial activity of copper against isolated evolved clones using standard ISO 22196:2011 testing, (iii) antibacterial susceptibility testing of evolved clones to ampicillin, chlorhexidine, ciprofloxacin, colistin, fosfomycin, gentamicin and trimethoprim, and (iv) whole-genome sequencing of evolved populations and isolated clones to obtain deeper insights into the molecular mechanisms and population dynamics underlying the evolution experiment. The developed method can be used to support the assessment of resistance risks related to AMCs during the development of novel materials in research and industry settings as well as during the authorization process by governmental authorities.

## Results

### Determining conditions for ALEE-AMC

**Fig. 1:**
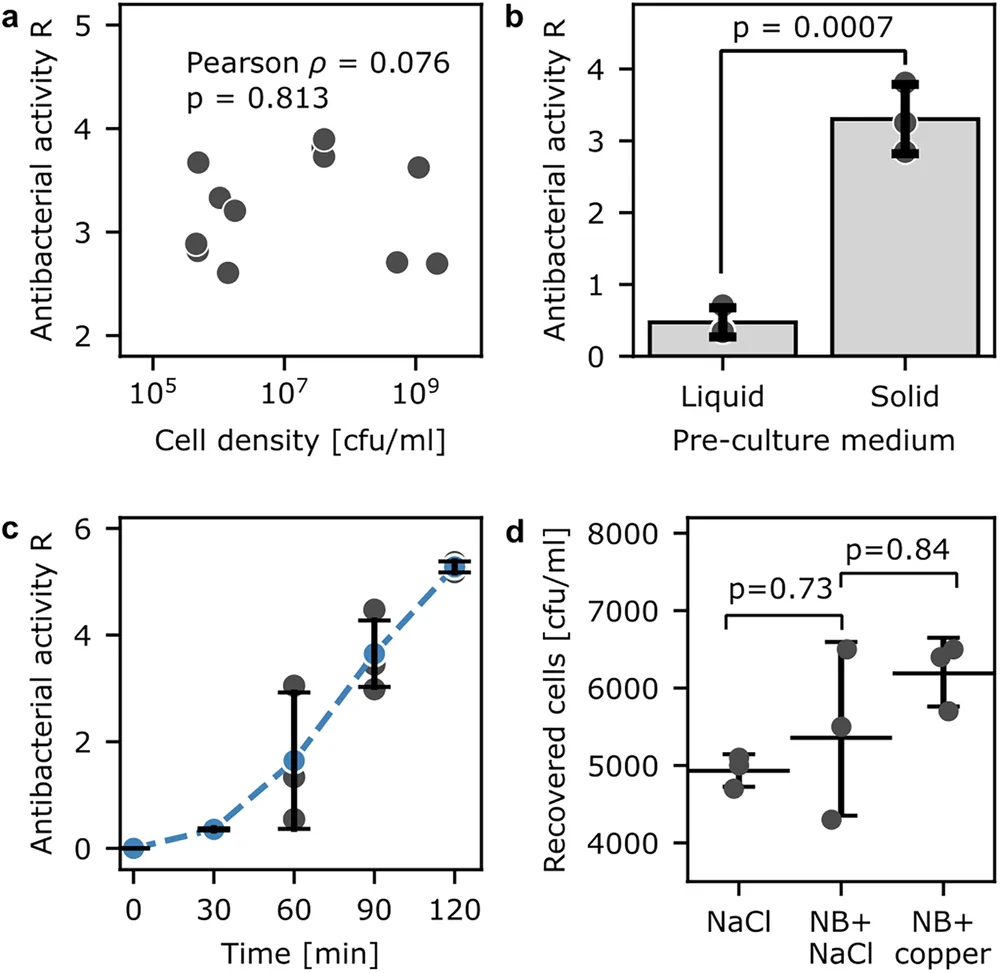
The effect of cell density, pre-culture conditions, exposure time, and neutralizer on antibacterial activity of metallic copper on *E. coli*. **a** Antibacterial activity (R) as a function of cell density. Cell inoculum was prepared from colonies grown on solid medium and exposed to copper for 30 min. No significant correlation between log-transformed cell density and antibacterial activity was observed (Pearson correlation coefficient *ρ* = 0.076, *p*-value = 0.813). **b** Antibacterial activity of copper depends on pre-culture conditions of the inoculum (*p* = 0.0007, two-sided two-sample *t*-test of *R*-values). Shown are *R*-values from experiments with an initial cell density of ~4 × 10^7^ cfu/mL (corresponding to ~1 × 10^6^ cfu/cm^2^) and 30 min of exposure. Bars represent the mean of *n* = 3 biological replicates, dots indicate individual replicates and error bars show the standard deviation. **c** Antibacterial activity of copper as a function of time at an initial cell density of 4 × 10^7^ cfu/mL (corresponding to ~1 × 10^6^ cfu/cm^2^). The inoculum was prepared from liquid cultures. Blue markers connected by the dashed line indicate the mean, gray dots represent individual replicates, error bars are standard deviations. The experiment was carried out with *n* = 3 biological replicates per timepoint. **d** Nutrient broth (NB) medium is a suitable neutralizer to stop the antimicrobial effect of copper according to the IBRG neutralizer test. Horizontal lines indicate mean values, gray dots are individual replicates, error bars are standard deviations of *n* = 3 biological replicates. *P*-values were obtained from one-sided two-sample *t*-test of log-transformed cfu/mL. A one-sided test was chosen because the IBRG neutralizer test asks to confirm that the tentative neutralizer does not lead to a reduction in cfu (i.e., a change of cfu in one direction).

An adaptive laboratory evolution experiment to be performed on AMCs (ALEE-AMC) was developed based on ISO 22196:2011, an international standard to determine the antimicrobial activity of non-porous solid surfaces. The evolution protocol consists of repeated cycles of exposure to an antimicrobial surface based on ISO 22196:2011 and cultivation of bacteria that survive the exposure. The protocol can select for mutants with elevated survival upon exposure to antimicrobial surfaces. Such mutants emerge spontaneously during cell growth in the cultivation phase in the absence of the AMC. Prior to executing ALEE-AMC with copper as reference AMC and *E. coli* as reference organism, critical parameters of the ISO 22196:2011 protocol were adjusted and experimentally investigated to be suitable for a cyclic exposure protocol. Adaptive evolution strongly depends on the population size (opportunity for mutants to emerge). Therefore, the cell density in the inoculum was increased from a target value of 6 × 10^5^ cfu/mL as used in ISO 22196:2011 to a target value of 2 × 10^8^ cfu/mL in the evolution experiment. The impact of cell density in the inoculum on the antibacterial activity (R) of copper was tested in a range from ca. 10^5^ to 10^9^ cfu/mL. No decrease in antimicrobial activity was observed when the cell density of the inoculum was increased within the above-stated range and exposed to copper for 30 min (Fig. 1a).

Experimental evolution for increased survival can be performed by alternating between killing and liquid growth cycles^32^, while ISO 22196:2011 requires an inoculum prepared from cells growing as a colony on solid medium. Since killing can be dependent on cell physiology^33^, the effect of pre-culture conditions (liquid vs. colony) on antibacterial activity was tested. The results show that cells from liquid pre-cultures were less susceptible to copper as compared to cells originating from colonies grown on agar (Fig. 1b). Since the cultivation cycles in the evolution experiment were chosen to be carried out in liquid medium, the time for a ~1000-fold initial killing of bacteria on copper was determined with an inoculum prepared from liquid cultures.

Next, an exposure time was determined which resembles in-use conditions that allow the survival of part of the exposed bacteria, which in turn would enable us to test for the potential occurrence and selection of mutants with increased survival. Therefore, an exposure time that approximately kills 99.9% (*R* = 3, corresponding to 1000-fold reduction) of exposed bacteria (pre-grown as liquid cultures) was determined. This condition (*R* = 3) is also in line with current BPR guidelines for the efficacy requirements of AMCs with desired disinfecting properties in, e.g., medical settings^11^. Thus, a change of this antibacterial activity towards increased survival upon evolutionary adaptation could render the AMC ineffective when used for this purpose. In turn, this change in antibacterial activity could be interpreted as an evolutionary adaption towards resistance to the AMC. Determining the time-kill dynamics on copper showed that exposure for 90 min resulted in an antimicrobial activity *R* = 3.65 (range: 2.99–4.47) (Fig. 1c).

Lastly, a cyclic evolution protocol requires the ability to harvest surviving cells after exposure to copper and inoculate them into fresh medium, allowing re-growth during which random mutations are generated. Thus, it is essential that the recovery medium neutralizes the antibacterial activity of copper but also does not interfere with recovery of viable cells. Ideally, cells are directly harvested in their growth medium to minimize stress induced by media fluctuations and avoid cell loss due to unnecessary washing or dilution steps between recovery and inoculation for re-growth (to remove the neutralizer solution). Since cells were, according to ISO 22196:2011, cultured in NB medium, we tested whether NB medium is a suitable neutralizer for the antimicrobial effect of copper. To this end, we followed the neutralizer test published by the IBRG. The data showed that incubating cells with NB medium does not lead to cell loss as compared with NaCl, confirming that NB medium does not, as expected, have a negative effect on cells (Fig. 1d). Importantly, incubating cells with NB medium pre-incubated with copper showed no loss of cells as compared to incubation with pristine NB medium, demonstrating that NB effectively stops the antimicrobial effect of copper (Fig. 1d).

### ALEE-AMC can determine evolutionary adaptation to copper

**Fig. 2:**
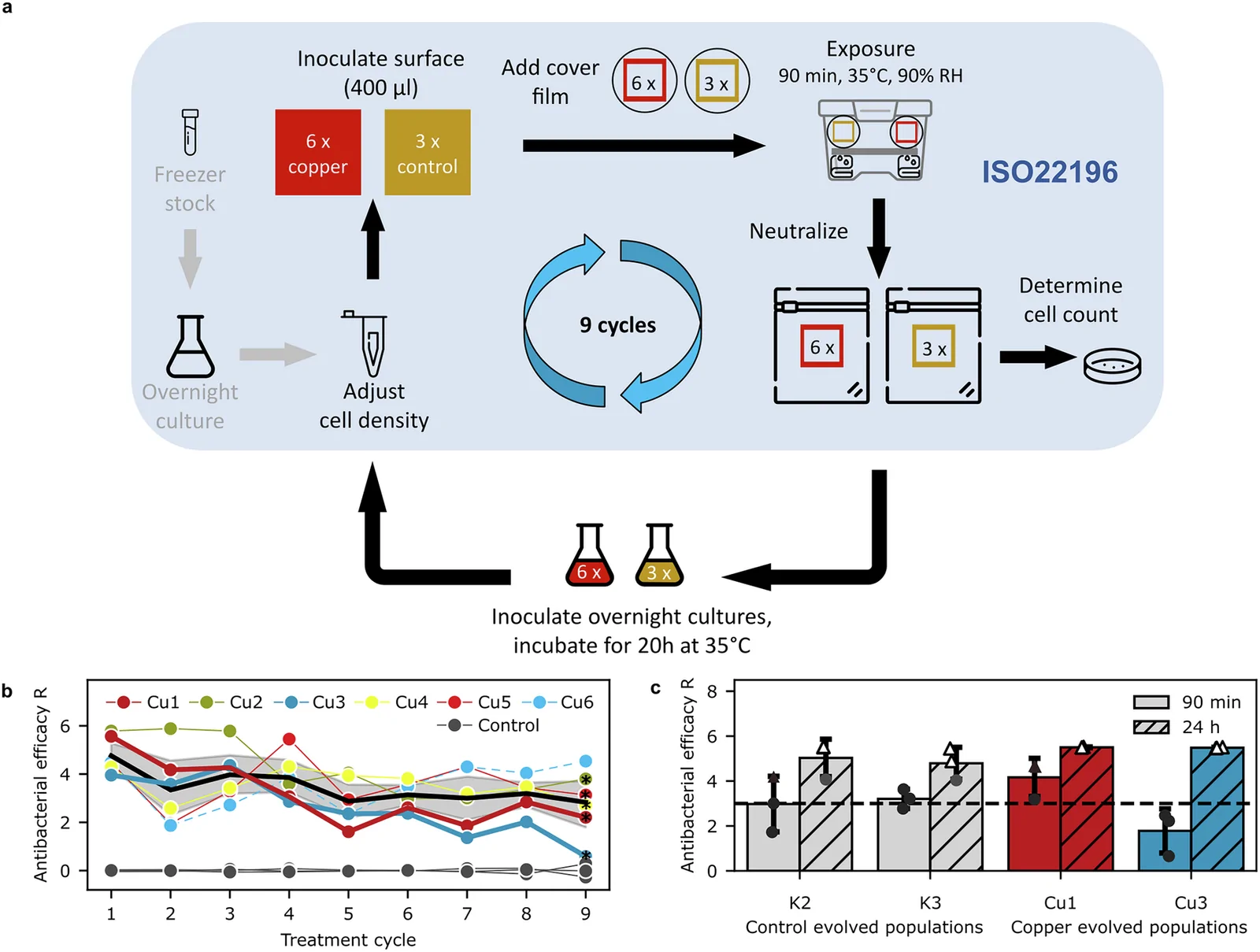
ALEE-AMC, a protocol to experimentally test evolutionary adaptation of bacteria to antibacterial surfaces and its application for evolution of *E. coli* after cyclic exposure to copper. **a** Schematic depiction of the ALEE-AMC method for experimental evolution, which is based on the international standard ISO 22196:2011 (blue box). RH – relative humidity. **b** Antibacterial activity of copper during ALEE-AMC performed at BAM. Black bold line shows the mean *R* over all copper-evolved replicates, gray area indicates the standard deviation. Solid lines and asterisks inside markers at ‘treatment cycle 9’ indicate a significant decrease of antibacterial activity after the final cycle (adj. *p* < 0.05 with Benjamini–Hochberg correction, One-sample *t*-test of *R*-value at cycle 9 versus *R*-values of all *n* = 6 populations at cycle 1). Populations highlighted in bold (blue and red) were used to isolate clones for subsequent testing of adaptation to copper. Dashed lines indicate no significant change in *R* at exposure cycle 9. Gray lines and circles correspond to populations that evolved on polymer control surfaces. **c** Testing of adaptation to copper of evolved clones according to ISO 22196:2011. Dashed line indicates *R* = 3. Colors correspond to parent populations in **b** from which clones were isolated. No statistical difference between clones from copper-evolved (blue and red) and control (gray) populations was detected for either the exposure time of 90 min or 24 h (adj. *p* < 0.05 with Benjamini–Hochberg correction, two-sided two-sample *t*-test on *R*-values). Open triangles indicate the limit of detection for cases in which no colonies were retrieved from the surface (see “Methods”). The figure contains elements (icons) used from flaticon.com.

ALEE-AMC was devised based on the international standard ISO 22196:2011 (Fig. 2a). Modifications to the standard were based on the experimental results shown in Fig. 1. ALEE-AMC was performed on six replicate populations which were cyclically exposed to copper surfaces and three control populations that were exposed to non-antimicrobial polymer surfaces. Cell counts of surviving cells were determined at each cycle after the exposure. The data showed that the antibacterial activity of copper during the first exposure cycle (mean *R* = 4.83, range 4 to 6) was slightly higher (*p* = 0.047, two-sided *t*-test of *R*-values) than the previously determined activity (*R* = 3.65, range: 2.99–4.47; Fig. 1 c), indicating variation of copper activity across experiments. We then evaluated whether evolved populations showed differences in antibacterial activity. To this end, we tested if the final *R*-value of each evolved population was different from the distribution of *R*-values at cycle 1, using a one-sample *t*-test. The results of cycle 1 can be interpreted as efficacy determination with 6 replicates, because each population has undergone a single round of growth and exposure. Hence, cycle 1 represents the baseline variance for the determination of *R*. Upon further passage, populations become independent, and therefore we test if the final *R*-value of each individual population is different from the distribution of *R*-values at cycle 1. For five out of the six replicate populations exposed to copper, a significant decrease in the antibacterial activity of copper was observed over the course of the experiment (Fig. 2b). Three evolved populations (Cu1, Cu3, Cu4) showed increased survival with an *R* < 3 (i.e., less than 3 log reduction of viable cells). Thus, these populations could be categorized as resistant to the AMC because their increased survival could render the AMC ineffective with respect to a disinfecting claim that requires a 3 log reduction according to BPR guidelines^11^.

After the last round of evolution, two populations originating from copper and control exposures were chosen (bold lines in Fig. 2b) for the isolation of individual clones and their adaptation with respect to survival on copper surfaces was assessed using the ISO 22196:2011 method (Fig. 2c). No statistically significant difference between control and copper-evolved clones in the antibacterial activity of copper after 24 h (as required by ISO 22196:2011) was observed (Fig. 2c). However, the antimicrobial activity at 24 h was very strong, leading to almost complete killing of the exposed cells. Therefore, an exposure time of 90 min was chosen and used instead throughout the evolution experiment. The data showed that one of the two clones (BAM-Cu3) exhibited a lower *R*-value at this exposure time, although no statistical significance to the control populations K2 and K3 was observed (Fig. 2c). This discordance between the data obtained from populations and those of individual clones indicates that the evolved populations were genetically diverse and that no single genotype with increased survival was fixed in the population.

### ALEE-AMC ring trial shows consistent adaptive response across laboratories

**Fig. 3:**
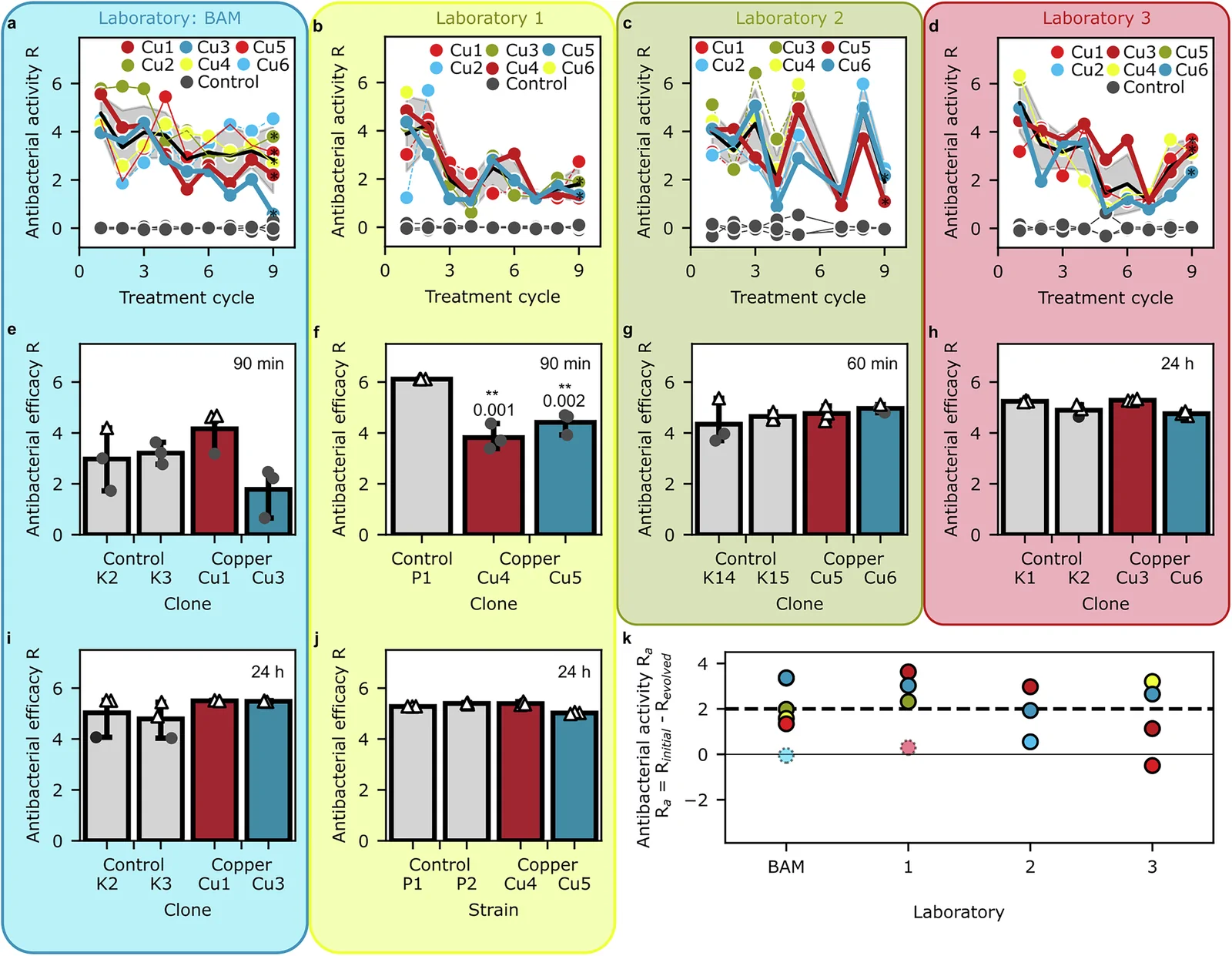
Results of the ring trial of ALEE-AMC with *E. coli* on copper. **a**–**d** Antibacterial activity over the time-course of the evolution experiment. Black bold lines show the mean *R* over all copper-evolved replicates, gray area indicates the standard deviation. Solid lines, asterisks inside markers at ‘treatment cycle 9’ indicate a significant decrease of antibacterial activity over the course of the experiment (*p* < 0.05 with Benjamini–Hochberg correction, One-sample *t*-test of *R*-value at cycle 9 versus *R*-values of all *n* = 6 populations at cycle 1). Exposure times to copper were determined by each partner and were chosen as follows: BAM: 90 min; laboratory 1: 35 min; laboratory 2: 60 min; laboratory 3: 60 min. Clones from populations with lines highlighted in bold (red and blue lines) were used for testing the adaptation regarding their survival on copper. Dashed lines indicate no significant change in *R*. Gray lines and circles correspond to populations that evolved on polymer control surfaces. **e**–**j** Testing for adaptation for survival of selected clones on copper according to ISO 22196:2011. The clones isolated from different populations highlighted in **a**, **b** have been tested after exposure for 90 min and 24 h. **e**, **i** correspond to populations with the same color code in **a**. **f**, **j** correspond to respective populations in **b**. **g** The clones isolated from populations in **c** were tested with exposure for 60 min. **h** The clones isolated from populations in **d** were tested with exposure for 24 h. Statistical significance of data in **e** to **j** was determined by two-sample *t*-test (*p* < 0.05) with Benjamini–Hochberg correction of *R*-values of copper-evolved versus control clones. Open triangles indicate the limit of detection for cases in which no colonies were retrieved from the surface (see “Methods”). **k** Adaptation of antibacterial activity (*R*_*a*_, Eq. 2) during the evolution experiment for populations observed in different testing laboratories (partners) as shown in **a** to **d** depicted with the same color code; zero indicates no change in antibacterial activity of copper after evolution. Positive values indicate decreasing antibacterial activity of copper after evolution. Negative *R*_*a*_ values indicate increased sensitivity to copper after evolution. Data points with low saturation and dashed edges correspond to non-significant replicate populations from **a**–**d** (test described under description of **a**). Data shown in **a**, **e**, and **i** are also shown in Fig. 2 and are displayed here again to enable direct interlaboratory comparison.

In order to investigate if the devised evolution protocol can produce consistent results across laboratories, a ring trial across six additional partners (excl. BAM) was conducted. Three partners executed the evolution protocol over the full nine cycles (Fig. 3b–d) and provided post-evolution characterization data and biological samples to perform the follow-up phenotypic characterization. One partner successfully conducted the evolution experiment but did not provide post-evolution characterization data and samples to perform the characterization. Two partners did not fully execute the evolution experiment due to technical challenges pertaining to calibration of the appropriate exposure conditions, failure to recover viable cells after the first round, or cross-contamination. In preparatory tests, each partner adjusted the exposure time to copper, aiming for an antibacterial activity *R* ≈ 3. This step showed that partners determined exposure times varying from 30 to 90 min and that initial *R* values were slightly higher than in the reference dataset (range of mean *R* between partners ranges between 3.8 and 5.3, Fig. 3a–d).

For all four laboratories (incl. BAM) that fully executed the experiment, most populations exposed to copper exhibited statistically significant increases in survival to copper after nine cycles (Fig. 3a–d), whereas other populations went extinct or did not show a change in survival on copper when comparing the *R*-values of the first and last cycle (Fig. 3a–d). All partners obtained populations that showed antibacterial activity of *R* < 3, which could be categorized as resistant with respect to a disinfecting claim according to BPR guidelines^11^ (see above). Each partner conducted the ISO 22196:2011 after ALEE-AMC with clones isolated from control populations which were exposed to polymer and isolated from evolved populations which exhibited increased copper-survival (Fig. 3e–j). No statistically significant changes in the antibacterial activity of copper after 24 h of exposure were detected. This suggests that adaptation to short exposure (35 to 90 min) does not select for survival for extended exposure durations. In one case, copper exhibited a significantly lower antibacterial activity against an evolved clone after exposure for 90 min (Fig. 3f). This indicates that the duration of the experiment was not long enough to enable stable fixation of clones with increased survival in all replicate populations for all partners.

To assess the results of ALEE-AMC across the four participating partners, the difference in the antibacterial activity of copper at the beginning and at the end of the experiment for each replicate population, the *adapted antibacterial activity R*_*a*_, was calculated and a one-way ANOVA was conducted on *R*_*a*_ values, using partner identity as factor (Eq. 2, and Fig. 3 k). This analysis showed no statistically significant difference between partners (*p* = 0.915), indicating that variation in *R*_*a*_ is not attributable to partner identity. Partner identity accounted for 3.77% of the total variance in *R*_*a*_.

Given that the results of ALEE-AMC are intended to be used for risk assessment, an alternative approach for data analysis is to consider the proportion of adapted populations as an indicator of the risk for adaptation. To distinguish adapted from non-adapted populations, a threshold for adaptation needs to be defined. We propose *R*_*a*_ ≥ 2 (i.e., at least a 99% decrease in antibacterial activity) to represent a meaningful and relevant adaptation to an AMC, given that current BPR guidelines recommend a threshold *R* ≥ 3 for relevant antimicrobial activity for surfaces with desired disinfecting properties. For each partner, the number of adapted (i.e., *R*_*a*_ ≥ 2) and non-adapted (*R*_*a*_ < 2) populations were determined, and logistic regression analysis was performed to test whether partner identity significantly influenced the number of replicates adapted above the set threshold. Again, no statistically significant effect between the results of different partners was observed (*p* = 0.6704). The approach of evaluating the results of ALEE-AMC in light of the number of adapted populations and the resulting implications for regulatory guidelines and statistical interpretation will be covered in the “Discussion” section.

Overall, the results of the ring trial displayed variability in the outcome of the evolution experiment both within and between partners. While it seems intuitive that this variability may be due to differences in external factors that pertain to the execution of the experiment at the level of individual partners (“lab-to-lab variation”, handling and treatment of samples, environmental factors such as temperature and humidity), no significant effect of partner identity on *R*_*a*_ was detected. Instead, intrinsic factors relating to, e.g., the non-specific mode-of-action of copper, the physiology of the bacterial cells, or the stochastic nature of evolution and the resulting evolutionary pathways towards adaptation appear to dominate the variability.

### Whole genome sequencing reveals multiple potential evolutionary pathways towards increased survival on copper surfaces

**Fig. 4:**
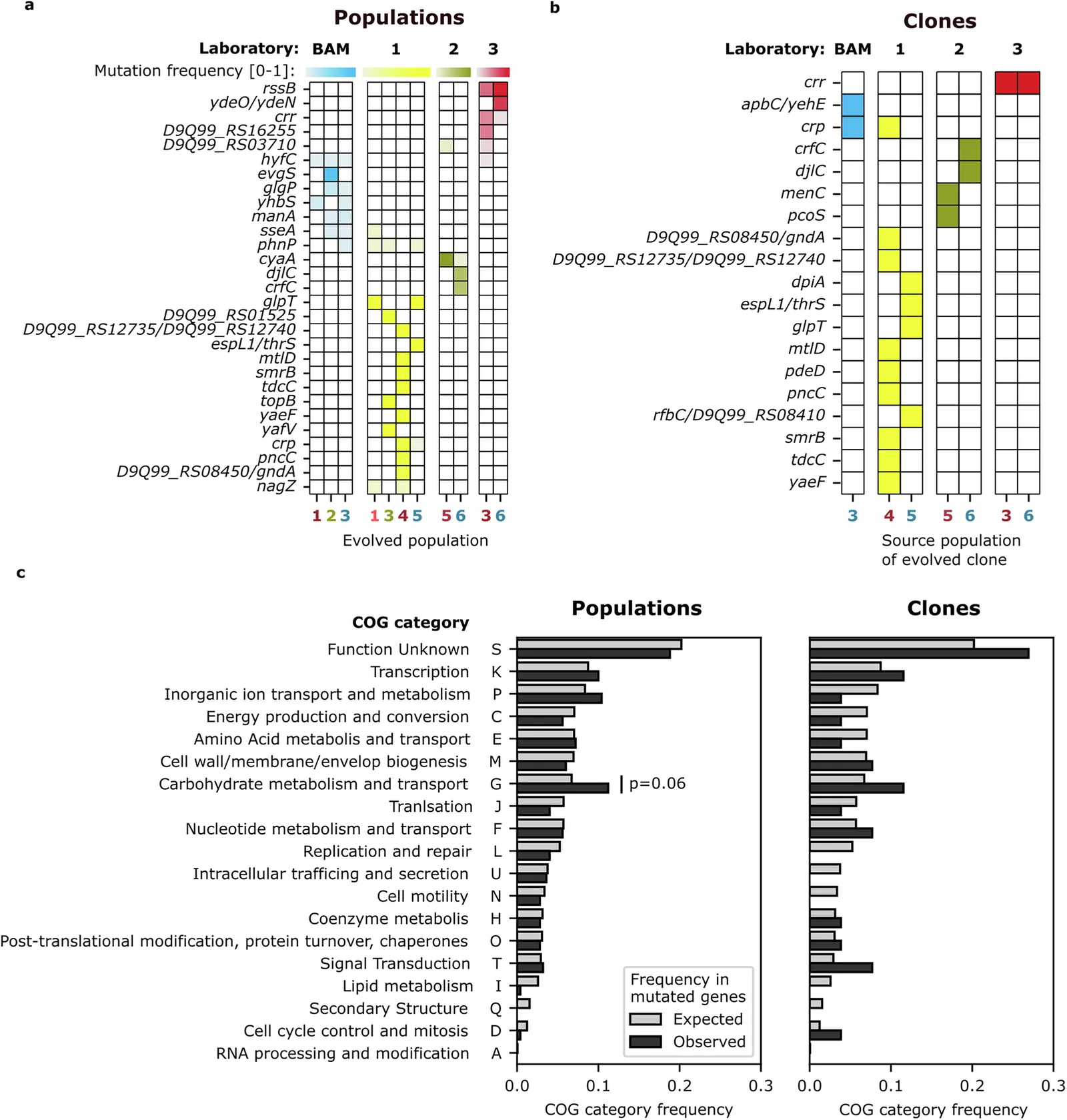
Whole genome sequencing reveals diverging evolutionary pathways towards increased survival on copper surfaces. **a**, **b** Mutations in populations and clones, respectively. Mutations in coding sequences and upstream of coding sequences are shown. Color-coding of red or blue labels is in correspondence to populations shown with Fig. 3. Shown are genes in which the summed frequency of all mutations in that gene are above 0.2 across the different sequenced populations. The color scale at the top of the panel indicates the frequency of the mutation. Mutations that occur in the populations and clones that evolved on polymer control surfaces have been subtracted from those occurring in copper-evolved clones and populations, establishing a list of mutations that are specific to the adaptation to copper surfaces. A list describing all mutations can be found in Supplementary Data S1. **c** Frequency of mutated genes belonging to different COG categories (dark gray) compared to expected frequency of genes in that COG category (light gray) in the genome of *E. coli* ATCC8739. According to Fisher’s exact test with Benjamini–Hochberg correction for multiple testing on the observed and expected gene counts per category, enrichment to *p* < 0.1 of COG category *G* ‘Carbohydrate metabolism and transport’ is indicated in the plot. No significant enrichment or depletion of genes mutated in the clones has been detected.

The genotypic changes that emerged upon exposure of *E. coli* to copper were analyzed by whole-genome sequencing. Several populations and clones per partner laboratory were selected for sequencing. Two approaches were followed; Firstly, genomic DNA for sequencing was extracted directly from the evolved populations re-grown after the last copper exposure, to gain an overview of the mutational spectrum. Secondly, individual clones from these populations were sequenced by picking colonies from agar plates. The latter approach allows linking mutations occurring within a single genotype, which is not possible with population sequencing. In addition, sequencing clones allows us to link genotypes to their phenotypes because we sequenced the same clones that were tested for their changes in copper survival (Figs. 2–4). Populations and clones from controls that were only exposed to polymers were also sequenced to determine mutations that might have occurred due to the general handling and cultivation over the course of the evolution experiment. Genes which acquired mutations in the promoter or coding region in the control conditions were subtracted from those occurring in copper-evolved clones and populations. Overall, mutations with a frequency above 5% were detected in the promoters or coding sequences of 201 unique genes in the control condition and in 179 unique genes in the copper condition (Supplementary Data S1).

The *E. coli* test strain ATCC8739 recommended in the ISO 22196:2011 standard harbors 61 IS mobile genetic elements, 20 of which are IS1 elements. These elements can cause genetic instability through frequent transposition events, which may partially explain the variability of the *R*-value of individual populations over time (Fig. 3a–d). In the clones, 29% of the mutations present after evolution on copper were associated with IS elements (6/21 mutations), three of which are predicted to disrupt gene function as the insertion site was within the coding sequence. Approximately 3% of all considered mutations in the copper-evolved population sequencing approach were associated with IS elements (6/202 mutations). Importantly, ATCC8739 carries a region with high homology to the *sil* and *pco* systems, which are associated with decreased susceptibility to silver and copper ions, respectively^28,31,34^.

Whole-genome sequencing revealed multiple evolutionary pathways upon stress caused by exposure to copper surfaces as a likely factor underlying the observed variability in the phenotypic assays (Fig. 4). Namely, there was little to no overlap of the mutated genes in the populations across laboratories (Fig. 4a, b). For example, populations evolved in laboratory 3 did not show a single mutated gene to overlap with populations evolved at laboratory 1. The same was true for populations evolved at BAM and laboratory 2. For the clones, there was only a single mutated gene (*crp*) that occurred twice across the seven sequenced clones. The data show that the evolved mechanisms in these diverging populations and clones must be underscored by different evolutionary pathways. Overall, mutations were found in several genes with a potential role in decreased susceptibility to copper, encoding for (i) copper resistance membrane-spanning protein (*pcoS*)^28^, (ii) metabolic genes related to phosphotransferase systems and carbohydrate transport (*crr, crp, cyaA, glpT*)^35,36^, (iii) the negative regulator of general stress response (*rssB*)^37^, and (iv) the co-chaperone DjlC involved in cadmium sensitivity (*djlC*)^38^. Mutations in the region between *copA* and *ybaQ*, coding for the  copper-exporting P-type ATPase CopA and a HigA family addiction module antitoxin, respectively, were also detected, but those occurred in copper-evolved populations along with control populations (Supplementary Data S1).

Next, we investigated whether mutations in genes with similar functions were overrepresented in the copper-evolved populations and clones. To this end, data from all partners were pooled and a functional enrichment analysis based on the observed and expected frequency of genes belonging to a given Cluster of Orthologous Genes (COG) was performed. To identify COG functional categories that were disproportionately affected by mutations, we performed a Fisher’s exact test for each category, comparing the number of mutated genes belonging to that category against the total number of mutated genes (observed frequency), relative to the composition of the full genome (expected frequency). This tests whether mutations are enriched in a given functional category beyond what would be expected by chance given the expected COG frequency across the genomic background. Similar approaches are frequently used to identify functional enrichment in genes stemming from, e.g., transcriptome data^39^ or genomic regions^40^. The analysis showed that genes associated with metabolism and transport were overrepresented in the mutated genes detected in the populations, as determined by functional enrichment analysis (Fig. 4c, d). Namely, genes annotated as belonging to COG category G "*Carbohydrate metabolism and transport”* were enriched (*p* = 0.06; Fisher’s exact test with Benjamini–Hochberg correction) in the populations as would be expected based on the frequency of this category in the ancestor genome. No significant enrichment was detected in the data relating to mutations in clones, which is likely limited by the low number of mutated genes in that dataset. These findings suggest that adaptation to copper exposure during ALEE-AMC was not associated with mutations in previously described copper stress response systems (such as PcoS, Cus, CopA or CueO) that have been shown to allow growth in the presence of elevated concentrations of copper ions^28,41^. Instead, mutations in metabolic genes were detected, which likely contributed to the increased survival in the presence of metallic copper.

### Adaptation to metallic copper is accompanied by the emergence of clinical resistance to fosfomycin and canonical resistance mutations

**Fig. 5:**
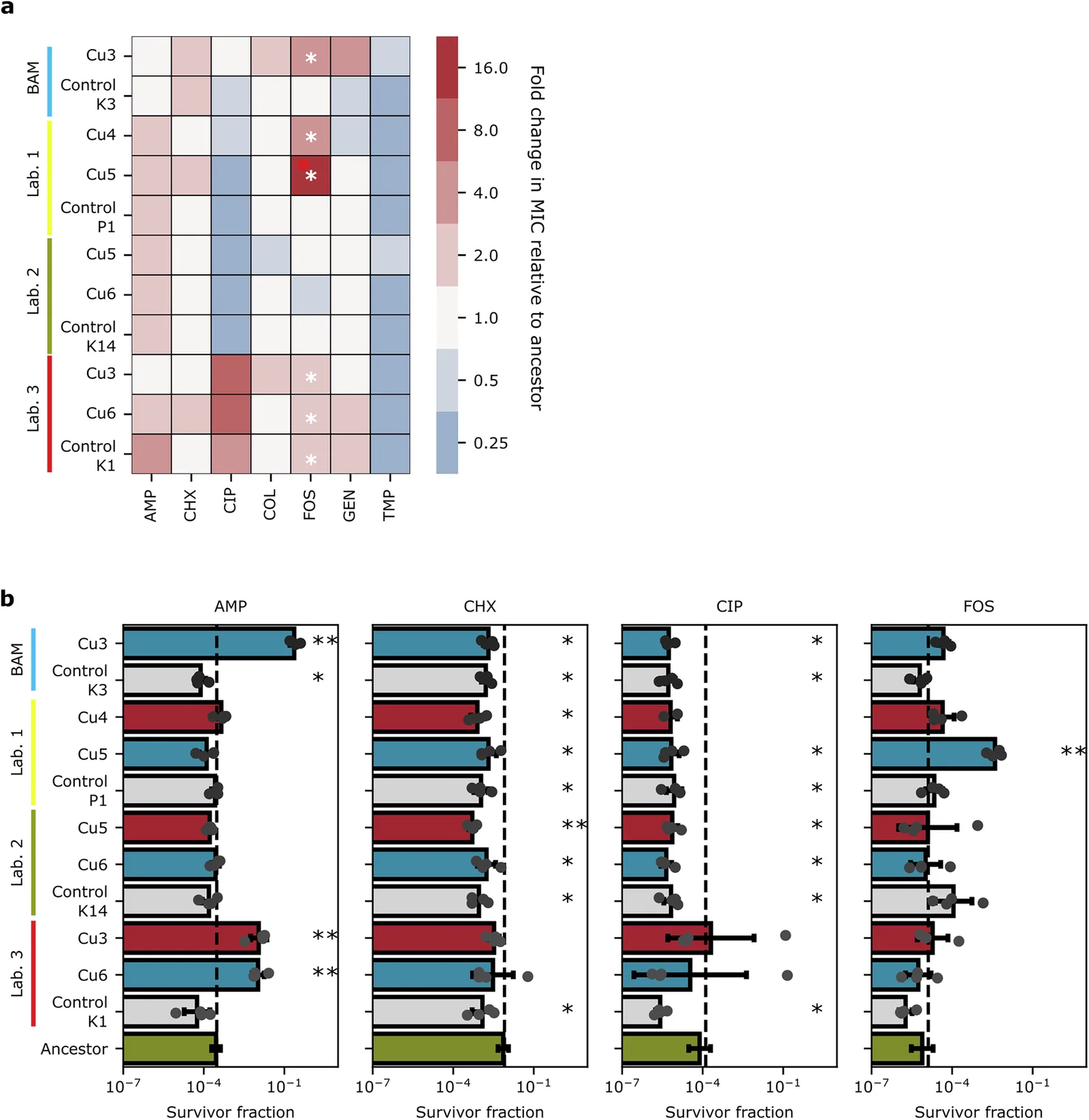
Cross-resistance and survival of copper-adapted clones against six antibiotics and chlorhexidine. **a** Cross-resistance determined by measuring the minimal inhibitory concentrations for copper-adapted clones relative to the ancestor. Susceptibility above the clinical breakpoint (CB) is indicated by asterisks. Biological replicates were *n* ≥ 2, whereby the highest determined MIC was used to calculate fold changes in case of variation between replicates. Ampicillin (AMP; MIC_ancestor_ = 4 µg/mL; CB_AMP_ = 8 µg/mL); chlorhexidine (CHX; MIC_ancestor_ = 0.48 µg/mL; no CB_CHX_ available); ciprofloxacin (CIP; MIC_ancestor_ = 0.0156 µg/mL; CB_CIP_ = 0.5 µg/mL); colistin (COL; MIC_ancestor_ = 0.25 µg/mL; no CB available; ECOFF_COL_ = 2 µg/mL); fosfomycin (FOS; MIC_ancestor_ = 8 µg/mL; CB_FOS_ = 8 µg/mL); gentamicin (GEN; MIC_ancestor_ = 0.125 µg/mL; CB_GEN_ = 2 µg/mL); trimethoprim (TMP; MIC_ancestor_ = 0.5 µg/mL; CB_TMP_ = 4 µg/mL). **b** Changes in survival were tested during exposure with 16 µg/mL AMP, 3 µg/mL CHX, 0.1 µg/mL CIP, or 16 µg/mL FOS for 3 h. Bars display the geometric mean survivor fraction, error bars the standard deviation, and circles the values of individual biological replicates (*n* = 4). The dashed lines indicate the mean survivor fraction of the ancestor strain. Statistical significance was determined through two-sided, unpaired *t*-test with Benjamini–Hochberg correction on the log-transformed survivor fraction of evolved clones versus the ancestor. Bar colors indicate copper evolved clone (red, blue), ancestor (green) or control evolved clone (gray). Red and blue bars correspond to source populations from Figs. 3 and 4. AMP, ampicillin; CHX, chlorhexidine; CIP, ciprofloxacin; COL, colistin; FOS, fosfomycin; GEN, gentamicin; TMP, trimethoprim.

Concerns have been raised that exposure of bacteria to antimicrobial surfaces can co-select for cross-resistance to antibiotics^4^. To investigate whether the mutations that occurred during evolution on copper lead to clinical antibiotic resistance, the minimal inhibitory concentration (MIC) of the evolved clones for six antibiotics (ampicillin, ciprofloxacin, colistin, fosfomycin, gentamicin and trimethoprim) from different classes was determined (Fig. 5a). In addition, one antimicrobial substance (chlorhexidine) was included in the analysis, which is used as either a disinfectant or an antiseptic. Surfaces of living tissue (e.g., skin) treated with chlorhexidine might be exposed to bacteria surviving on antimicrobial copper surfaces, e.g., in hospital settings, and it is therefore important to understand whether copper exposure can co-select for decreased chlorhexidine susceptibility. The results show that the changes in MICs ranged from a fourfold decrease to a sixteen-fold increase for most of the clone-antimicrobial combinations tested (Fig. 5a). Ampicillin and chlorhexidine displayed exclusively increased MICs across different clones, while trimethoprim displayed decreased MICs. In turn, the MICs to ciprofloxacin, colistin, fosfomycin, and gentamicin increased or decreased depending on the clones tested. The changes in MIC for most of the clones were below the clinical breakpoints of the antibiotics tested and therefore do not represent cases of potential clinical resistance evolution. However, six clones exhibited a MIC for fosfomycin above the clinical breakpoint of 8 µg/mL, whereby this was evident for one out of four tested clones that evolved on control surfaces and five out of seven clones that evolved on copper. Strikingly, clone Cu5 from laboratory 1 exhibited a MIC ≥ 128 µg/mL to fosfomycin. This clone had, in addition to three other mutations, a single base pair deletion at position 843/1359 in the coding sequence of *glpT* (Fig. 4b and Supplementary Data S1), which was also fixed in population 5 from which the clone was isolated. In addition, population 1 of laboratory 1 also showed a fixed mutation in *glpT* through the insertion of an IS1 element in the coding region (Fig. 4a). The gene *glpT* encodes the glycerol-3-phosphate transporter GlpT, which is known to be involved in fosfomycin uptake. Loss of function of GlpT is known to convey fosfomycin resistance^36^. Together, the results show that ALEE-AMC followed by antibiotic susceptibility testing can detect changes in MIC, including those above the clinical breakpoint. The data also suggest that adaptation to copper can be accompanied by clinical fosfomycin resistance. However, testing of considerably more clones is necessary to establish a sufficient level of statistical power to test for a link between adaptation to AMCs and antibiotic resistance (see “Discussion” for more details).

Metabolic adaptations, especially in phosphotransferase systems, have been associated with increased survival to several antimicrobials, including disinfectants, antiseptics and antibiotics^35^. Since mutations in these genes were widespread across clones, four antimicrobial agents (ampicillin, chlorhexidine, ciprofloxacin and fosfomycin) were selected and tested for the survival of the clones at lethal concentrations above the clinical breakpoint of the antibiotics and a lethal concentration for chlorhexidine (which does not have a clinical breakpoint) (Fig. 5b). The fosfomycin-resistant clone Cu5 (laboratory 1) exhibited >500-fold increased survival after 3 h exposure with 16 µg/mL fosfomycin (Fig. 5b). Clones Cu3 (BAM), Cu3 (laboratory 3) and Cu6 (laboratory 3) exhibited elevated survival after 3 h exposure with ampicillin at 16 µg/mL. All three clones carried mutations in *crr* or *crp*, both of which are related to phosphotransferase systems. The mutation found in *crp* for clone Cu3 (BAM) was in a region important for binding of cAMP, suggesting that it might directly affect the function of CRP^42^. Clones Cu3 (laboratory 3) and Cu6 (laboratory 3) both carried IS1 insertions in the coding sequence of the glucose phosphotransferase gene *crr*. However, clone Cu4 (laboratory 1) did not show changes in ampicillin survival despite having a mutation in *crp* (Fig. 5b). Clone Cu4 (laboratory 1) carried an additional 8 mutations, which might affect the phenotype. Interestingly, none of the clones, which exhibited elevated survival to ampicillin, had a notable increase in the MIC of ampicillin. This is in line with previous findings suggesting that mutations in metabolic genes may affect survival without changing resistance (which requires the ability to grow at increased concentrations)^32,35^. Another interesting observation was that almost all clones isolated from copper or control populations exhibited a slight, but statistically significant, decrease in survival against chlorhexidine and ciprofloxacin (on average 78% and 85% reduced survival, respectively) compared to the ancestor. In conclusion, cross-resistance to fosfomycin and increased survival in the presence of ampicillin and fosfomycin were predominantly detected in copper-adapted populations and were accompanied by mutations in metabolic genes. Future work could focus on testing more clones to test for a statistical link between adaptation on AMCs and antibiotic resistance and on establishing causal relationships between the identified mutations and the observed phenotypes.

## Discussion

Currently, a standardized test to assess evolutionary adaptation of bacteria in response to AMCs is lacking. In this paper, we present such a standardizable test, ALEE-AMC, to assess the potential of adaptive evolution within bacteria in response to AMCs towards increased survival. ALEE-AMC is based on the well-established international standard for testing the activity of non-porous antimicrobial surfaces, ISO 22196:2011. ALEE-AMC can be implemented in laboratories which already routinely perform ISO 22196:2011 testing. We established the protocol, along with a reference dataset, using the protocol in a ring trial with copper as reference AMC and *E. coli* as reference organism. The protocol has been designed to be readily implemented in the normal schedule of commercial testing laboratories that operate with regular working hours from Monday to Friday, which fosters its uptake into practice. It is important to note that ALEE-AMC determines the *potential* for adaptation and its consequences for cross-resistance to antibiotics. It does not predict the rate of evolution and the evolved mechanisms that might occur in real life because the test format does not mimic real-life conditions for many applications of AMCs, and the test is performed with only one surrogate organism. It is known that environmental conditions and strain genetic background strongly affect evolutionary trajectories^43^. Many AMCs are designed to be used under dry or semi-dry conditions, which are different to the wet conditions used in ISO 22196:2011 and ALEE-AMC test formats. While currently a few methods exist that aim to mimic exposure under dry or semi-dry conditions^44–48^, these tests do not have the level of simplicity, reproducibility and international coverage as ISO 22196:2011. At the same time, these methods do not operate with a sufficiently high bacterial population size to enable experimental evolution, and they have limited throughput.

During the development of ALEE-AMC, several key parameters of the ISO 22196:2011 method were modified to make the method suitable for assessing the potential of bacteria to evolve increased survival to AMCs due to repeated exposure. To this end, the influence of cell physiology, cell density of the inoculum, and copper exposure time on the antibacterial activity of copper has been determined (Fig. 1). Changes in exposure time and cell physiology had strong effects on the antibacterial activity of copper (Fig. 1b, c). Thus, it has been decided that the determination of an exposure time to achieve a pre-defined antibacterial activity must be part of ALEE-AMC. The activity was aligned with regulatory guidelines that require a 3 log reduction of viable cells within 60 min (copper required 35 to 90 min in our hands) for an AMC with a disinfecting claim in medical settings testing^11^. Loss of this efficacy could be categorized as resistance to the AMC. Furthermore, it is conceivable that variations in exposure time and cell physiology can affect antibacterial activity and thereby the selective pressure exerted on the populations during the evolution experiment. In the reference dataset generated here (Fig. 2), variations in the antibacterial activity between populations on the same exposure cycle, but also within populations on multiple exposure cycles, were observed (Fig. 2). Besides differences in exposure time and cell physiology, which could dominate the variation between laboratories (Fig. 3), the inherent randomness with which mutations occur and their subsequent effect on selection dynamics within populations likely contribute to the observed variability. While experimental parameters can, to an extent, be controlled, variation from evolutionary processes must be considered when evaluating the experimental results (discussed in more detail below). Despite the observed variability within laboratories, the endpoint measurements indicate that the antimicrobial activity of copper against most evolved populations had substantially declined (Fig. 3k), which demonstrates that ALEE-AMC is a suitable method to assess adaptation of bacteria to AMCs.

Mechanistic analysis showed that no mutations in genes known to be involved in copper homeostasis and detoxification were detected in the evolved *E. coli* clones and populations, except for one clone with a mutation in *pcoS* (Fig. 4). The only other observed mutation related to metal resistance was in *djlC*, which is annotated to play a role in cadmium sensitivity. Instead, mutations in genes involved in the general stress response and carbon metabolism were frequently detected (Fig. 4). Previous studies in which bacteria evolved on copper showed similar results in terms of mutated genes^29–31^. Several factors can explain these findings: First, different traits are important for growth in the presence of copper ions in solutions as compared to traits that are important for short-term survival of high-copper stress exerted by copper surfaces, and which were selected for in ALEE-AMC. For example, it is well documented that bacteria can evolve to exhibit several 100-fold higher survival in the presence of antibiotics and disinfectants without showing substantial changes in their ability to grow in the presence of these substances^32,49^. In the context of antibiotics, it has been speculated that mutations that increase survival are more frequent than mutations that increase the ability to grow at elevated concentrations^8^. While some genes have previously been associated with increased survival to one or multiple stressors (e.g., *rssB, cyaA, crp, crr, glpT*), it has proven difficult to directly link mutations that arise in response to lethal metal stress to the survival phenotype^29–31^. Bleichert et al.^29^ interpreted the transcriptomic state of copper-adapted clones to be similar to that of stressed cells in a “shallow persister” state. This highlights the possibility that the effect of diverse mutations converges on a similar physiological state conferring high copper survival, which could be assessed by transcriptomics, proteomics or metabolomic analysis.

The evolution on copper resulted in changes in the antimicrobial susceptibility of individual clones, even though not all the investigated strains showed changed survival on copper. In addition to altered survival in the presence of lethal concentrations of antibiotics (Fig. 5b), the most striking observation here is the selection of a highly fosfomycin-resistant strain with a MIC well above the clinical breakpoint. This strain carried a mutation in the canonical fosfomycin-resistance determinant *glpT*. In addition, a different mutation in *glpT* was detected at high frequency in another population (Fig. 4b, and Supplementary Data 1). In light of the global antimicrobial resistance crisis, this highlights the need to investigate the evolution and selection dynamics that occur on AMCs and consider their potential to select for cross-resistance to antibiotics. However, further studies are needed to directly link the altered susceptibility to the mutations in *glpT*. The data also support the notion that any test for evolutionary adaptation to AMCs should be followed by antimicrobial susceptibility testing against antibiotics using standardized protocols and comparing the obtained changes in susceptibility to clinical breakpoints. Such tests should be done with isolated clones, regardless of changes in their susceptibility to the AMC. However, it should be noted that more isolated clones must be tested to establish a sufficient level of statistical power to either show or exclude an association between evolution on AMCs and antibiotic resistance. A simple power analysis based on Fisher’s exact test shows that with a likelihood of occurrence of ca. 70% in the treatment group (similar to the 5 resistant clones observed out of 7 copper-evolved clones tested) versus 25% in the control group (similar to 1 observed out of 4 tested) requires testing of about 18 clones to reach a statistical power of 80%. The required likelihood of occurrence that should be monitored with ALEE-AMC should be agreed upon by regulators and risk assessors in the future in order to establish a meaningful level of tested clones from the evolved populations.

The selective pressures acting during experimental evolution are not always obvious and have to be carefully considered in the experimental design^50^. In ALEE-AMC, the two main forces driving selection are the ability to survive copper exposure and the ability to reproduce rapidly in the absence of copper. Decreased susceptibility to antimicrobial substances is often associated with decreased maximal growth rate^32,51^. Very strong trade-offs between both traits could result in highly dynamic changes in populations, which might result in fluctuations in their ability to survive copper stress, alternating between genotypes with fast growth and genotypes with high survival. Such effects could contribute to the temporal fluctuations of survival observed along the trajectories of the evolution experiments (Figs. 2 and 3). Additionally, increased survival will result in a higher starting cell number in the next growth phase, which can consequentially change cell physiology, for instance by altering the time that the population spends in stationary phase. In turn, this changed physiology can affect the survival during the subsequent exposure to copper (Fig. 1).

Little parallelism was observed between mutations from different laboratories, which could be due to differences in killing strength, selection dynamics, differences in laboratory conditions, as well as a large number of potential mutations that increase copper survival, and the randomness of mutation in general. Very strong bottlenecks might result in genetic drift and enrich “hitchhiker” mutations, which are random and not directly associated with the selective pressure in the experiment. These effects might vary depending on the AMC that is being tested. The inherent randomness of mutations that drive evolution poses an important challenge when the risk for evolutionary adaptation is to be assessed.

In order to use ALEE-AMC as a standard, we suggest the proportion of replicates which adapted their survival above a pre-defined threshold after repeated exposure to the antimicrobial surface as the characteristic parameter to be reported. Regulatory bodies could then define a threshold for the proportion of adapted replicates beyond which adaptation to the tested surface is deemed unacceptable. First, this requires a criterion for scoring a replicate as “adapted”, i.e., it displays a relevant increase in survival (decrease in *R*). Here, we propose *R*_*a*_ ≥ 2 as such a relevant change in *R*, i.e., a 99% reduction in antimicrobial activity. Second, a threshold for the proportion of adapted replicate populations is required. In the following, we discuss a statistical method that links the choice of the threshold with the number of required replicates for the test and the observed adaptive outcomes. By making the distinction between adapted and non-adapted replicates, binomial statistics can be used to evaluate the outcome of the evolution experiment and to determine the number of replicates required in an evolution experiment. For instance, if a threshold frequency of adapted replicates (i.e., proportion of adapted replicates) is defined to be 0.4 by a regulatory body, it can be calculated that at least 6 replicates are required in an experiment in which none may display adaptation to conclude that the frequency of adaptation is significantly lower than 0.4 (Table 1). If adaptation is to be observed in one replicate, a total of 10 replicates is required to arrive at the same conclusion. Table 1 summarizes threshold adaptation frequencies and the required number of replicates if zero or one adapted replicate shall be observed.

**Table 1:** Minimal number of required replicates in ALEE-AMC for different regulatory adaptation frequency thresholds

Adaptation frequency threshold^a^	Minimum No. of replicates (no adaptation in any of the replicates)^b^	*p*-value^c^	Minimum No. of replicates (with one adapted replicate)^d^	*p*-value^c^
0.2	14	0.044	22	0.048
0.3	9	0.040	14	0.048
0.4	6	0.047	10	0.046
0.5	5	0.031	8	0.035
0.6	4	0.026	6	0.041
0.7	3	0.027	5	0.031
0.8	2	0.04	4	0.027

^a^Adaptation frequency threshold of a tested bacterial strain on a certain antimicrobial surface. The threshold needs to be set prior to the experiment.

^b^Minimum number of replicates required that show no adaptation. For instance, to confirm an adaptation frequency threshold ≤0.4 with a significance level >95% (*p* = 0.047), an experiment with 6 replicates needs to be performed, in which no adaptation is observed.

^c^*p*-values were calculated with a binominal test.

^d^Minimum number of replicates required that have one adapted replicate. E.g., to confirm an adaptation frequency threshold ≤0.4 with a probability of 0.046, an experiment with 10 replicates needs to be performed, in which adaptation of one replicate is observed.

A question that arises is how to define a meaningful threshold for the adaptation frequency. The threshold for newly developed surfaces could be informed by reference data, such as the outcome of the ring trial on copper presented here. In the ring trial, no significant effect of partner identity on *R*_*a*_ could be detected, and we can calculate the adaptation frequency from the 8 replicates out of 24 that achieved *R*_*a*_ ≥ 2. Hence, we assume that the true frequency of adaptation of *E. coli* to copper is 0.33 (95% CI: 0.16–0.55, assuming binomial statistics). If the claim is to be made that the frequency of adaptation of the new surface is less than a threshold of 0.33, no adapted line may be observed in a total of at least 8 replicates (*p* = 0.039; binomial test). The statistical power and the number of required replicates of this approach will depend on the difference between the threshold set for the frequency of adaptation and the true frequency of adaptation (Table 1). Defining a meaningful threshold might prove challenging unless reference data are available.

The work presented here demonstrates that adaptation of bacteria to AMCs can be quantified by applying already standardized test formats and that such experiments can result in consistent evolutionary outcomes. However, several challenges need to be overcome as next steps towards standardization, mainly pertaining to the choice of reference AMC and reference bacterial strain. These two factors are key to allowing for a reference dataset that can consistently be reproduced by any test laboratory accredited to apply the standard. The chosen *E. coli* strain (ATCC 8739, DSM 1576), which is required in ISO 22196:2011, harbors numerous IS elements which are very active under stressful environmental conditions. These IS elements could be related to the observed heterogenous adaptation. Therefore, it would be advisable to identify a different strain of *E. coli* that shows more homogenous adaptation related to randomly occurring single nucleotide polymorphisms rather than large-scale genomic rearrangements^32^.

The AMC used as reference for the test needs to have (i) a consistent antimicrobial activity with little sample-to-sample variation, (ii) an antimicrobial activity towards the reference strain that allows the convenient determination of a specific effect (3 to 4 log_10_ decrease) within a manageable incubation time (≥30 min), and (iii) possibly consistent molecular mechanisms that underpin the adaptation of the reference strain. However, our data show that the combination of copper with *E. coli* (ATCC 8739, DSM 1576) does not provide ideal characteristics towards these requirements. *First*, the surface chemistry of copper strongly depends on the quality of the material, the manufacturing conditions and the environmental conditions of storage, and this in turn affects its antimicrobial activity. Specifically, copper can have various oxidation layer compositions depending on the environmental conditions, which have strong effects on its antimicrobial activity^52^. The composition of the oxidation layer might depend on manufacturing conditions (chemicals used for production or heat introduced during the cutting of coupons), environmental conditions during storage (humidity, light, temperature), and further treatment of the surface (polishing); all of which are difficult to standardize across laboratories. Indeed, our data show that the time to obtain a given antimicrobial effect of copper varies between laboratories despite using the same source of copper and the same bacterial strain (Fig. 3). *Second*, the rather strong antimicrobial effect of copper results in the notion that small changes in exposure may strongly impact the resultant killing observed. This could explain the within- and between-lab variation between replicates and could lead to uneven selective pressure within a single evolutionary line across different transfer cycles. *Third*, the genomic data of the evolution experiment reported here (Fig. 4) showed heterogenous mechanisms underlying evolutionary adaptation across replicate populations. It has been shown previously that adaptation to quaternary ammonium compounds (QAC) in solution can result in highly parallel evolution^32^. Future research could test whether QAC-based antimicrobial coatings^53^ can also support such parallelisms in adaptation. Taken together, future experiments could use the approach established in this study to identify a strain-AMC combination that could serve as a consistent reference for the procedure to be established across different laboratories. Once a testing laboratory can reproduce adaptation with the reference AMC-strain combination, the procedure can be applied to any AMC that needs to be tested. The choice of the reference AMC-strain combination should consider homogenous AMCs, reproducibility and repeatability of a measurable but sufficiently strong antimicrobial effect, and parallelism in the adaptive response of the bacterial strain.

Overall, political and regulatory decisions should be taken on whether the results of a test measuring the evolutionary adaptive potential of microorganisms to antimicrobial surfaces are required for authorization. Such considerations should weigh the costs associated with obtaining the data against the risks associated with adaptation, leading to loss of efficacy of AMCs or clinically relevant cross-resistance to antibiotics. In comparison to other standards that determine antimicrobial susceptibility to antibiotics or disinfectants, the test is rather complex, and consequently, it is estimated to be about 15 to 20 times more expensive than conventional testing with ISO 22196:2011 despite its duration being limited to 9 exposure cycles. This duration is relatively short compared with experimental evolution protocols used in academic settings. Indeed, we observed that those 9 cycles were not enough to fix the mutants in the population but were enough to show relevant adaptation to decreased antibacterial activity of the AMC. In contrast, previous studies with QACs in solution have shown that ~10 cycles can be sufficient to obtain fixation in the population given the strong selective pressure exerted by a killing effect of 3 to 4 log_10_^32^. Moreover, scientific arguments should be provided to regulatory bodies to justify an unacceptable adaptation frequency threshold. Ultimately, it should be in the interest of manufacturers and end users of AMCs to quantify the potential for adaptation to their product in order to sustainably maintain its efficacy and desired protective effect.

In summary, we developed a standardizable cyclic exposure method to investigate the adaptation of bacteria towards increased survival on AMCs caused by de novo mutations followed by selection. The method is based upon the internationally recognized standard for efficacy testing (ISO 22196:2011). The results show consistent adaptation in terms of increased survival on copper for evolved populations between participating laboratories, but not for a limited number of isolated clones. The difference in survival between the initial and evolved antibacterial activity, quantified by the parameter *R*_*a*_, has been suggested as the output of the test, which can be analyzed by binomial statistics to determine the potential for evolution towards the loss of efficacy of the AMC. Downstream analysis provided insights into the molecular mechanisms underlying adaptation to copper, the parallelisms of mutations within and between laboratories, and the consequences of adaptation to copper for cross-resistance to antibiotics. Future improvements of the method should focus on the choice of reference strains and the reference AMC, allowing more reproducible antimicrobial activity and a higher parallelism in the adapted genes in order to produce a reference dataset, which can be implemented as a positive control in different laboratories that aim to perform the test with novel AMCs.

## Methods

### Materials

Two types of non-porous test surfaces were used: coupons consisting entirely of metallic copper (>99.90% Cu, UNS-Nr.: C11000, CAS: 7440-50-8, CEN/TS 13388:2015-08: CW004A, Trade name: Wieland-K32, Wieland Werke, Germany) and control coupons made from polyurethane (Pur D44 Shore 80° hardness grade A, Reiff, Germany). Coupons of both surfaces had a size of 50  × 50 mm with a thickness of 2 mm. Prior to use, the coupons were placed for 15 min in 70% ethanol and then dried under sterile conditions. Prior to exposure to bacteria, the test coupons were pre-conditioned for at least 1 h in a humid chamber set to 35 °C to ensure consistent temperature and humidity. Polymer control coupons were reused for different experiments, while copper coupons were used only once. Polymer control coupons were cleaned in LabDet 100 (MED00003, Meintrup DWS, Germany) for 24 h, rinsed with deionized water, and air-dried. A detailed description of materials and their preparation for experiments is provided in Supplementary Information in Sections 2.2 and 3.2.

### Media, microorganisms and culturing

The microorganism *E. coli* (ATCC 8739, DSM 1576) used in this study was ordered from the German Collection of Microorganisms and Cell Cultures (DSMZ, Braunschweig, Germany). All media were prepared in deionized water (conductivity of <1 μS/cm), adjusted to a pH of 7.0–7.2 and sterilized in a high-pressure steam autoclave (121 °C for 20 min) prior to use. Bacterial cells were cultured at 35 °C as required by ISO 22196:2011 for 16 to 24 h either as liquid culture at 150 rpm orbital shaking in 10 mL nutrient broth medium (NB) composed of 3 g/L meat extract (Sigma, PO 1.03979-500 G), 10 g/L peptone (Roth, PO 8986.1) and 5 g/L sodium chloride (Chemsolute, PO 1367) by inoculating 10^2^ cells from a freshly-thawed freezer stock or cells grown on plate count agar (PCA) composed of 2.5 g/L yeast extract (Roth, PO 2363), 5 g/L tryptone (Serva, PO 48647), 1 g/L glucose (Merck, PO 49159) and 15 g/L agar (AppliChem, PO A0949). PCA was inoculated by dilution-streaking cells from a liquid culture with an inoculation loop. A 1:500 dilution of NB medium in water (1/500 NB medium) was used to adjust the cell suspension to the desired cell density prior to testing the antibacterial activity of test surfaces. Phosphate-buffered saline (PBS) was prepared by establishing an 800-fold phosphate-buffered stock solution consisting of 34 g/L potassium dihydrogen phosphate (PO 1648) set to a pH 7 ± 0.2 with NaOH. PBS was then prepared by mixing 1.25 mL of this stock solution with 100 mL tenfold physiological saline solution consisting of 85 g/L NaCl and adding deionized water to 1000 mL. PBS was used to establish a dilution series of bacterial cell suspensions before spotting or plating on PCA plates. Agar plates were incubated at 35 °C for ca. 18 h to determine the viable cell number by counting colony-forming units (CFU). Freezer stocks were prepared by growing an overnight culture in NB medium to OD_600_ ~ 2–6 and mixing it with 60% glycerol at a 3:1 ratio (0.75 mL culture and 0.25 mL 60% glycerol). A detailed description of media and culture conditions is provided in Supplementary Information Sections 2.3, 3.1 and 3.3.

### Neutralizer test

The neutralizer test was performed according to the test method P16/001/1.01 Annex IV published by the International Biodeterioration Research Group (IBRG)^54^. An *E. coli* culture was grown overnight in NB medium. The culture was adjusted in PBS to reach ~10^3^ cells/mL upon dilution of 1 mL of this cell suspension into the following three experimental conditions: (A) 10 mL 0.85% NaCl solution, (B) 9 mL neutralizer solution (NB medium) plus 1 mL 0.85% NaCl, and (C) 10 mL neutralizer solution (NB medium), containing a copper coupon pre-incubated for 30 min. The cells were incubated under these three conditions at room temperature for 30 min. Thereafter, samples were diluted in PBS and plated on PCA, and then incubated at 35 °C overnight to assess the number of CFU from each test condition. The role of the neutralizer solution is to quench the antibacterial activity of the test surface. The neutralizer solution is regarded as suitable if similar numbers (tested with students *t*-test at *α* = 0.05) of CFU are recovered from conditions A and B (i.e., if the neutralizer itself is non-toxic) and if there is less than 50% reduction (defined in P16/001/1.01) observed in condition C as compared to A (i.e., the neutralizer solution quenches the antibacterial activity of the test surface). The test showed that NB medium is a suitable neutralizer for copper (Fig. 1) and was therefore used as neutralizing agent during all subsequent experiments.

### Antibacterial activity of the test surfaces

The antibacterial activity of the copper surfaces and the polymer control surfaces against the test microorganisms was initially evaluated following the ISO 22196:2011 standard. An overnight culture was adjusted in 1/500 NB medium to a defined viable cell count to establish a cell suspension for later exposure. Adjustments were performed either by counting cells under an optical microscope in a Thoma counting chamber or by measuring optical density at 600 nm (OD_600_) with a photometer, and always validated by plating appropriate dilutions of the adjusted cell suspension on PCA followed by counting the number of cfu that developed subsequently. For surface exposure, 400 µL of adjusted cell suspension was applied to sterile test coupons that were placed in individual Petri dishes with vents. Each of the inoculated coupons was then covered with a sterile plastic film and incubated at 35 °C in a humid chamber placed in a temperature-controlled incubator for defined time intervals. The humid chamber consisted of a sealed box filled with wet paper cloth, reaching ~90% relative humidity. Each test included 3 to 6 biological replicates per test surface. At the end of the exposure period, each of the test coupons was neutralized in 10 mL NB medium in a sterile stomacher bag by removing the plastic cover film and manually rubbing the surface in the neutralizing solution. Viable cells recovered from the test coupons were serially diluted in PBS and plated onto PCA either by spotting 10 µL or by plating 100 µL. A detailed description of the procedures for antibacterial activity determination is provided in Supplementary Information Sections 3.4 and 3.5.

The antibacterial activity (*R*) was calculated according to ISO 22196:2011 based on the difference in CFU after exposure to an untreated polymer control after exposure to copper.

1 $${{\rm{R}}}={{{\rm{U}}}}_{{{\rm{t}}}}{ - }{{{\rm{A}}}}_{{{\rm{t}}}}$$

, where *U*_*t*_ is the common logarithm of the number of viable bacteria (in cells/cm^2^) recovered from the untreated control surface at the end of the exposure period averaged over all untreated test coupons, and where *A*_*t*_ is the common logarithm of the number of viable bacteria (in cells/cm^2^) recovered from the antibacterial test surface at the end of the exposure period. The upper limit of detection (LOD) of antibacterial activity *R* depends on the cell number before exposure, the cell number on the control surface after exposure, the volume of the neutralizer, and the lower limit of detection of the plating method. Therefore, the upper LOD of *R* may vary between samples. For samples from which no cells could be recovered after exposure to the copper surface (i.e., CFU counts below the detection limit of the plating method), the upper LOD of *R* was calculated and used for further calculation of mean, standard deviations and statistical tests. Individual samples that reached the LOD are indicated in each figure as open triangles. A detailed description of the calculations for antibacterial activity determination is provided in Supplementary Information Section 4.1.

Antibacterial activity experiments were performed with different pre-culture conditions, different cell densities in the adjusted cell suspension and different exposure times (shown Fig. 1a–c). *Different pre-culture conditions* were either established on PCA plates as described in ISO 22196:2011 or with cells grown in NB medium, both of which were adjusted to ~4 × 10^7^ cells/mL and exposed to copper for 30 min. Experiments with *different cell densities* were performed by adjusting an overnight culture grown in NB medium to cover the range specified by ISO 22196:2011 ~10^5^ cells/mL (specifically 2.5–10 × 10^5^ cells/mL), and then up to ~10^7^ cells/mL and ~10^9^ cells/mL. The cells were exposed to copper for 30 min. *Different exposure times* were tested by performing a time-kill experiment with cells grown in NB medium adjusted to ~4 × 10^7^ cells/mL. To this end, parallel test coupons were prepared as described above to have replicates (*n* = 3) for each of the desired timepoints (0; 30; 60; 90 and 120 min). All test coupons were inoculated at the same time with the same cell suspension, placed in the incubator and three replicates were sacrificed at each timepoint. A detailed description of determining the exposure time for the evolution experiment is provided in Supplementary Information Section 3.6. Together, the experiments showed that colony-grown cells have a different survival than liquid-grown cells, that the tested cell densities do not limit the antibacterial activity of copper, and that 90 min exposure of a liquid-grown culture leads to an antibacterial activity (*R*) of ~3 (Fig. 1). Thus, these conditions were chosen for the evolution experiment described below.

### Determining evolutionary adaptation with ALEE-AMC and copper susceptibility testing of evolved populations

The adaptive evolution experiments (Fig. 2a, Supplementary Fig. S1, and Supplementary Information Section 3.7.1) were started by preparing an inoculum from a single overnight culture grown in NB medium adjusted to 2  × 10^8^ cells/mL (range from 0.2 to 6 × 10^8^ cells/mL) in 1/500 NB. 400 µL aliquots of this cell suspension were exposed to six replicate copper surfaces and three replicate polymer control surfaces at 35 °C for 90 min as described in section “Antibacterial activity of the test surfaces”. The polymer control surfaces account for any adaptation occurring as a result of the test procedure, excluding the antimicrobial effect of the copper surfaces. The number of control polymer surfaces was reduced compared to the copper surfaces because pre-experiments reproducibly showed no change in survival across transfers, and this reduction helps to minimize the future costs of the developed standard. A sample of the inoculum cell suspension was diluted in PBS and plated on PCA to determine the initial cell number (*A*_0_). Three test coupons were inoculated with 400 µL cell suspension at once and then moved directly to the incubator to maintain an exact exposure time and to minimize disturbance of the incubation chamber. The exposure was stopped by placing each test coupon into a separate sterile Stomacher bag filled with 10 mL NB medium as neutralizer in order to manually remove the surviving bacteria. A sample from the Stomacher bag was withdrawn to determine the number of surviving cells (*A*_*t*_) by plating appropriate dilutions on PCA as described above. In addition, 1 mL of the recovered cells were transferred from each Stomacher bag into a separate Erlenmeyer flask pre-filled with 9 mL NB medium, resulting in an overall 250-fold dilution of the initial 400 µL cell suspension exposed to copper after each exposure cycle. The transferred cells were incubated in an orbital shaker at 35 °C for 16 h to 24 h at 150 rpm. The overnight cultures were again used for exposure to freshly prepared test coupons, as described above. The maximum OD_600_ of the *E. coli* strain used in NB medium was determined before the start of the evolution experiment. During the evolution experiment, the OD_600_ of each overnight culture was measured to ensure that they had reached the stationary phase before exposure to copper. The above-described exposure to copper and control surfaces was repeated for 9 cycles for each replicate population. The procedure allowed us to perform three exposures and growth cycles per standard working week (Tuesday, Wednesday, Thursday). After the 3^rd^ and the 6^th^ cycle, the overnight cultures were stored at 4 °C for 3 days (i.e., from Friday to Monday). These cultures were then used to inoculate a fresh overnight culture on Monday by adding 100 µL to 10 mL fresh NB medium, followed by incubation at 35 °C for 16 h to 24 h.

The antibacterial activity of each population at each transfer cycle was calculated according to Eq. 1. The change in antibacterial activity due to *adaptation* (*R*_*a*_) was assessed for each evolutionary population according to

2 $${{\rm{R}}}_{{\rm{a}}}={{\rm{R}}}_{{\rm{i}}} - {{\rm{R}}}_{{\rm{e}}}$$

where *R*_i_ is the *initial* antibacterial activity of copper after the first exposure cycle (at the start of the evolution experiment) and *R*_*e*_ is the *evolved* antibacterial activity of copper at the end of the evolution experiment (after the 9^th^ transfer). Freezer stocks were prepared from overnight cultures of each of the evolved populations each day as described in section “Media, microorganisms and culturing”. A detailed description of the calculations relating to the evolution experiment is provided in Supplementary Information Sections 4.2–4.4.

### Isolation of evolved clones and testing their adaptation to copper

ALEE-AMC resulted in six populations that evolved on copper surfaces and three populations that evolved on polymer control surfaces. The different evolutionary lines are likely to be populations composed of a mixture of different clones that emerged by random mutation and that then underwent natural selection according to the environmental conditions of the assay, including copper stress. Individual clones were isolated from the evolved populations to test for the emergence of clones with increased survival. Clones were isolated from two evolved populations for which copper showed the lowest evolved antimicrobial activity (*R*_*e*_) of all replicate populations. In addition, the adaptation in the evolved population chosen for clone isolation should show at least a tenfold increase in survival as compared to the first exposure cycle (i.e., *R*_*i*_ ≥ *R*_*e*_ + 1). Moreover, two clones each from a different control population exposed to polymer coupons were isolated.

To isolate the clones from the last treatment cycle, the liquid overnight cultures were appropriately diluted, plated on PCA, and incubated overnight at 35 °C. In some cases, clones were isolated from freezer stocks by thawing the freezer stock, appropriate dilution, and plating on PCA. From the resulting plates, one or two colonies were re-streaked again on PCA. A single colony from each plate was used to inoculate an overnight culture in NB medium followed by incubation at 35 °C with orbital shaking at 150 rpm. The resulting overnight cultures were used to inoculate a pre-culture for testing adaptation of this clone to copper. In addition, the overnight culture was used to store a freezer stock as described above. In total, the isolation procedure resulted in five transfers without copper stress (one growth cycle in NB medium directly after the last copper exposure; two growth cycles on PCA for isolation and purifying the colony; two growth cycles in NB medium to prepare the cell suspension of the clone for copper exposure). Thus, the isolation procedure serves as a stability passage, increasing the chance that any change in antibacterial activity of the isolated clone is due to heritable genetic changes that emerged during the evolution experiment. A detailed description of sampling and storage of evolved cultures and clones is provided in Supplementary Information Section 3.7.2.

Adaptation of the clones isolated from the polymer control and copper-exposed populations was tested as described in section “Antibacterial activity of the test surfaces” at a cell density of ~4 × 10^7^ cells/mL in the exposed cell suspension. Two exposure times to copper were chosen. First, the exposure time of the evolution experiment (i.e., 90 min) and second, the exposure time required by ISO 22196:2011 (i.e., 24 h). The antibacterial activity (*R*) according to Eq. 1 was calculated for each clone. A detailed description of determining the adaptation of evolved clones is provided in Supplementary Information section 3.8.

### Ring trial

Six additional, independent laboratories across Europe (two from Germany, two from the UK, one from France, and one from Estonia) agreed to contribute to a ring trial of the method. The results were blinded for the presentation of the results. Detailed instructions were provided to the participants: a draft standard describing the method (similar to Supplementary Information), a detailed step-by-step protocol supporting the implementation of the experiment, a video file showing the handling of the samples, instructions to send clones and DNA for sequencing, and an Excel sheet to record the colony count data. In addition, the participants were provided with the following materials to conduct the test: an aliquot of the *E. coli* test strain (DSM 1576) to ensure the same genomic composition of the starting culture, a sufficient number of copper coupons (Wieland-K32, Wieland Werke, Germany), control coupons made from polyurethane (Pur D44 Shore 80° hardness grade A, Reiff, Germany), sterile plastic films to cover the copper coupons during exposure of the bacterial culture, and LabDet 100 (MED00003, Meintrup DWS, Germany) to clean the polymer control coupons.

The participants were asked to work in three steps: (i) Condition testing; i.e., to determine the exposure time that leads to *R* ≈ 3 (Eq. 1) in their laboratory by measuring a time-kill curve for *E. coli* on copper inoculated from a liquid culture as described in section “Antibacterial activity of the test surfaces”. The estimated duration of effort was approximately 1 week. (ii) ALEE-AMC with six replicate populations exposed to copper and three replicate populations exposed to polymer control, using their determined exposure time as described in section “Determining evolutionary adaptation with ALEE-AMC and copper susceptibility testing of evolved populations”. The estimated duration of effort was approximately three weeks. (iii) Testing of isolated clones for their adaptation to copper as described in section “Isolation of evolved clones and testing their adaptation to copper”. The estimated duration of effort was approximately one week. Participants were asked to isolate and test at least two clones from control populations and two clones from the copper-exposed populations with the lowest *R*_*e*_-value. Initially, participants were asked to perform testing of adaptation to copper at an exposure time of 24 h according to the original ISO 22196:2011. However, initial results from partners (laboratory 3) showed that 24 h exposure led to complete killing. Therefore, testing at 24 h exposure was unlikely to reveal differences in adaptation of clones regarding increased survival, which were selected at much shorter exposure times used during the evolution experiment. Thus, it was decided during the already ongoing trial to ask laboratories to perform testing for survival of clones at the exposure time determined by them (as done by laboratory 2). The clones provided by laboratory 1 were tested together with the clones from BAM with an exposure time of 90 min.

In addition, participants were asked to send samples for DNA sequencing from evolved populations (those from which clones were isolated) mixed with DNA/RNA shield (R1100-50, Zymo Research) instead of glycerol. In addition, participants were asked to send living isolated clones using the agar stab method on PCA originating from a sub-sample from the pre-cultures used for testing adaptation to copper (i.e., after approximately five stability passages). These samples were used for DNA isolation from clones and antimicrobial susceptibility testing.

### Antimicrobial susceptibility testing: determination of minimal inhibitory concentrations and ability to survive exposure to antimicrobials

Antimicrobial susceptibility testing was performed according to the guidelines of EUCAST using the ISO 20776-1^55^ and *E. coli* (ATCC 25922, DSM1103) as a reference strain. Testing was performed for the ancestor strain (ATCC 8739, DSM 1576) and for isolated clones (control and copper-exposed) from all participants of the ring trial. Testing was performed for the following antibiotics: ampicillin (Roth, K029), ciprofloxacin (Sigma-Aldrich, 17850), colistin (Sigma-Aldrich, C4461), fosfomycin (Sigma-Aldrich, P5396), gentamicin (Sigma-Aldrich, G1272), and trimethoprim (Sigma-Aldrich, PHR1056). Fosfomycin testing was performed by adding 25 µg/mL glucose-6-phosphate to the cultures. In addition, susceptibility testing was also performed for the disinfectant and antiseptic chlorhexidine (MP Biomedicals, 219136025). The following concentration (in µg/mL) panels were used: ciprofloxacin (0.0039; 0.0078; 0.0156; 0.0313; 0.0625; 0.125; 0.25; 0.5; 1; 2), ampicillin, gentamicin, colistin, trimethoprim (0.0156; 0.0313; 0.0625; 0.125; 0.25; 0.5; 1; 2; 4; 8), fosfomycin (0; 0.25; 0.5; 1; 2; 4; 8; 16; 32; 64), and chlorhexidine (0; 0.12; 0.24; 0.48; 0.96; 1.92; 3.84; 7.68). Susceptibility testing for chlorhexidine deviated from ISO 20776-1, as it was performed in NB medium instead of Mueller-Hinton (MH2) medium. Resistance to antibiotics was scored according to clinical breakpoints for *Enterobacterales* published by EUCAST (Clinical Breakpoint Tables v. 15.0)^56^.

The ability of evolved clones to survive exposure to lethal concentrations of antimicrobials was tested by determining the survival of the clones against ampicillin (16 µg/mL), ciprofloxacin (0.1 µg/mL), chlorhexidine (3 µg/mL), and fosfomycin (16 µg/mL) plus 25 µg/mL glucose-6-phosphate at an exposure time of 3 h to the respective antimicrobial. Replicate cultures of each strain were inoculated into 200 µL NB medium in a 96-well plate (Greiner) and incubated at 35 °C with shaking for 16 to 24 h. Cultures were then diluted 1:200 in 600 µL NB in a 1.5 mL 96-deep-well plate (LABSOLUTE) and incubated at 35 °C with shaking to an OD_600_ between 0.1 and 0.2. From each well, 100 µL culture was transferred to a 96-well plate containing 100 µL NB with twofold-concentrated antimicrobial. After 3 h incubation at 35 °C with shaking, samples were taken from each well, serially diluted and spot-plated on PCA. CFUs per milliliter after antimicrobial exposure were normalized to cfu/mL prior to exposure to yield the survivor fraction.

### Whole genome sequencing and bioinformatic analysis

DNA extraction for whole genome sequencing was performed from evolved populations directly after re-growth in NB medium of cells that survived the last copper exposure cycle described in section “Determining evolutionary adaptation with ALEE-AMC and copper susceptibility testing of evolved populations”. DNA extraction of isolated clones was performed directly from the pre-culture that was used for testing adaptation to copper (see section “Isolation of evolved clones and testing their adaptation to copper”). Thus, these clones had been exposed to a stability passage of five growth cycles in the absence of copper stress. Genomic DNA was isolated using the Zymo Quick DNA Kit (Zymo Research) after removal of DNA/RNA shield and lysis with lysozyme. Whole genome sequencing of the ancestor strain was performed on an Illumina MiSeq in 2×250 base pair (bp) paired-end mode, yielding at least 0.3 million read pairs, which corresponds to approximately 30-fold coverage of the *E. coli* genome (provided by MicrobesNG (https://microbesng.com, Birmingham, UK). Whole-genome sequencing of evolved clones and populations was performed on an Illumina NovaSeq 6000 in 2×150 bp paired-end mode, yielding at least 5 million read pairs per sample, which corresponds to approximately 300-fold coverage of the *E. coli* genome (provided by Eurofins Genomics Germany). The sequence data have been deposited in NCBI under Bioproject number PRJNA1438693.

Variant calling was conducted with breseq^57^ using the *E. coli* ATCC8739 genome as reference (NCBI accession: NZ_CP033020.1). In the sequence of the ancestor strain used here, nine differences from the deposited reference genome were detected. Because the focus here was on mutations that were selected by copper exposure, mutations that were present in the ancestor strain and mutations that were present in the control populations and clones exposed to polymer were considered not to be specific for the adaptation to copper. Genes which acquired mutations in the promoter or coding region under control conditions were subtracted from the copper-evolved clones and populations, establishing a list of mutations that are specific to the adaption to copper surfaces for further downstream analyzes. Biological interpretation of sequencing results was greatly aided by EcoCyc database^58^. Insertion sequence (IS) elements in the ancestor strain were detected by the software ISEScan^59^. Functional annotation of the ATCC8739 genome to obtain COG categories for each gene was done using eggnog-mapper^60^.

### Statistical analysis

Unless stated otherwise, statistical analysis was conducted in Python 3.11, using the Python packages scipy.stats and statsmodels^61,62^. Statistical tests and their corresponding parameters applied to specific data are specified in the respective figure captions. Pearson correlation was computed using stats.pearsonr. A two-sided two-sample *t*-test was performed with stats.ttest_ind(alternative = 'two-sided'). One-sided two-sample *t*-test was performed with stats.ttest_ind(alternative = 'greater). Fisher’s exact test was performed using stats.fisher_exact(). Binomial testing was performed with stats.binomtest(alternative = 'greater'). Benjamini–Hochberg correction for multiple testing was performed using stats.false_discovery_control(method='bh'). For logistic regression, the dependent variable (Outcome) was coded as binary based on the adaptation threshold (i.e., *R*_*a*_ ≥ 2) and non-adapted (*R*_*a*_ < 2) with laboratory identity (Lab) included as categorical predictor variable. Logistic regression was then performed with statsmodels.formula.api.logit('Outcome ~ C(Lab)'). One-way ANOVA was performed with statsmodels.formula.api.ols('R_a_ ~ C(Lab)').

## Supplementary information

Supplementary information

Supplementary information

## Data Availability

DNA sequences generated here can be found in NCBI under BioProject number PRJNA1438693. All other data generated during this study are included in this article and its Supplementary Data files.
