# Supplementary material for: ALEE-AMC: a standardizable method to determine evolutionary adaptation of bacteria on non-porous surfaces with antibacterial activity
Source: NPJ Antimicrob Resist. 2026 Nov 12;4:80. doi: 10.1038/s44259-026-00297-2 (PMC13759535; doi:10.1038/s44259-026-00297-2)
Supplement: Supplementary file 1 [file 44259_2026_297_MOESM1_ESM.pdf]

Supplementary Information

Related to

ALEE-AMC: a standardizable method to determine evolutionary adaptation of bacteria on non-porous surfaces with antibacterial activity

Niclas Nordholt[a,*,g], Selina B. I. Schmidt[a,*,h], Viola Boenke[a,*], Claudia Sündermann[a], Susann Wiltner[a], Beatrice Anger[b], Charlotte Valentin[b], Arnaud Bridier[b], Christophe Soumet[b], Alexander J. Cunliffe[c], James Redfern[c], Gillian Iredale[d], Peter D. Askew[d], Diana C. Mogrovejo[e], Marina Ruhlandt[e], Jan-Hendrik Klock[e], Florian H. H. Brill[e], Merilin Rosenberg[f], Angela Ivask[f], and Frank Schreiber[a#]

[a]Division of Biodeterioration of Materials (4.1), Department of Materials and the Environment, Federal Institute for Materials Research and Testing (BAM), Unter den Eichen 87, 12205 Berlin, Germany
[b]Antibiotics, Biocides, Residues and Resistance unit, French Agency for Food, Environmental and Occupational Health & Safety (Anses), Fougères Laboratory, Bâtiment Bioagropolis, 10 B rue Claude Bourgelat, CS40608, Javené, 35306 Fougères Cedex, France
[c]Department of Natural Sciences, Faculty of Science and Engineering, Manchester Metropolitan University, Chester Street, Manchester, M1 5GD, UK
[d]Industrial Microbiological Services Limited (IMSL), Pale Lane, Hartley Wintney, Hants RG27 8DH, UK
[e]Microbiology Department, Dr. Brill + Partner GmbH Institute for Hygiene and Microbiology, Grützmühlenweg 44, 22339 Hamburg, Germany
[f]Institute of Molecular and Cell Biology, University of Tartu, Riia 23, 51010, Tartu, Estonia
[g]present address: Department of Biochemistry, Charité – Universitätsmedizin Berlin, Berlin, Germany
[h]present address: SLB, Sandsliåsen 40, 5254 Bergen, Norway
*these authors contributed equally

Supplementary note

Description of Supplementary-Data-S1

Supplementary_Data_S1.xlsx: Mutations found in populations and clones following the evolution on copper or control surfaces.

Sheet 1: Mutated genes in copper lines in populations and clones

Sheet 2: Mutated genes in control lines in populations and clones

Sheet 3: Mutated genes only in copper in populations and clones (genes which acquired mutations in the promoter or coding region in the control conditions were subtracted from those occurring in copper-evolved clones and populations)

Sheet 4 – Data to plot Fig. 1a

Sheet 5 - Data to plot Fig. 1b: cfu/ml

Sheet 6 – Data to plot Fig. 1b: With calculated *R*-values

Sheet 7 – Data to plot Fig. 1c

Sheet 8 – Data to plot Fig. 1d

Sheet 9 – Data to plot Fig. 2b

Sheet 10 – Data to plot Fig. 2c

Sheet 11 – Data to plot Fig. 3a,b,c,d

Sheet 12 – Data to plot Fig. 3a,b,c,d: *p*-values of one-sample t-test of *R*-value at cycle 9 versus *R*-values of all populations at cycle 1

Sheet 13 – Data to plot Fig. 3e,f,g,h,i,j

Sheet 14 – Data to plot Fig. 3k

Sheet 15 – Data to plot Fig. 5a

Sheet 16 – Data to plot Fig. 5b

Supplementary text

Draft standard: Determination of evolutionary adaptation of bacteria on non-porous surfaces with antibacterial activity

1. Introduction

The determination of antibacterial activity on plastics and other non-porous surfaces is specified by the ISO22196:2011 which was prepared by the Technical Committee ISO/TC 61, *Plastics*, Subcomittee SC 6, *Ageing, chemical and environmental resistance*. The ISO22196:2011 is designed to assess the antibacterial activity of test coupon by determining the log fold reduction of bacterial cells of specific bacterial strains at a defined inoculum after 24 h incubation.

Materials with antibacterial activity can exert a selection pressure on microorganisms and thus may lead to the evolution of resistance. Since antibacterial resistance is becoming one of the major concerns for public health, the risk of materials with antibacterial activity for resistance evolution should be assessed.

The scope of this potential standard is to determine the evolution of resistance by *de novo* mutation followed by selection of bacteria exposed to materials with antibacterial activity. The procedure is described for determining the effect of copper on *E. coli* as a reference but can be applied to other non-porous surfaces. The acquired data should ultimately allow the estimation of the risk of adaptive evolution. The method uses the ISO 22196:2011 and extends it to an adaptive laboratory evolution experiment.

2. Materials

2.1. Bacterial strains to be used for the tests

a) *Escherichia coli* (ATCC 8739, DSM 1576)

2.2. Test coupons

The test can be conducted with any non-porous material. The test material should have antibacterial activity as determined by ISO22196:2011. In addition, a material with non-antibacterial material should be included as negative control. Copper is suggested as an antibacterial reference surface (positive control) and polyurethane polymer plates as negative control. Test coupons should ideally have the dimension of 5 cm × 5 cm.

	Copper<br>UNS-Nr.: C11000<br>CEN/TS 13388:2015-08): CW004A<br>CAS: 7440-50-8<br>Trade name: Wieland-K32	Polymer (untreated)<br>Trade name: PUR-plates<br>D44 80° Shore
**Size**	50 mm x 50 mm	50 mm x 50 mm
**Thickness**	2 mm	2 mm
**Purity**	> 99.90 % Cu<br>< 0.040 % O	Polyurethan
**Supplier**	Wieland-Werke AG<br>Graf-Arco-Straße 36<br>89079 Ulm, Germany	-
**Antibacterial activity ISO22196:2011 24 h**	$R_{(E.\ coli)}$ = 5.57	$R_{(E.\ coli)}$ = <0.1

2.3. Reagents, culture media and solutions

All culture media and solutions shall be prepared in deionized water (conductivity of < 1 µS/cm) and sterilized in a high-pressure steam autoclave (121 +/- 2°C for 20min) prior to usage. All reagents shall be of analytical grade and/or of a grade appropriate for microbiological purposes.

2.3.1. Culture media

NB medium

Compounds	CAS	1 L	PO[1]	Supplier
Meat extract	68990-09-0	3 g	1.03979-500G	Sigma
Peptone (caseine)	91079-40-2	10 g	8986.1	Roth
Sodium chloride	7647-14-5	5 g	1367	Chemsolute

[1] the listed products are example products that were used to acquire the reference dataset

Adjust the pH of the NB medium to 7.0 - 7.2 with NaOH or HCL and sterilize by autoclaving. Store in the fridge at 5°C-10°C and use within one month after preparation.

1/500 NB medium

Dilute NB media 1:500 (e.g. 2 mL NB on 998 mL deionized water or 1 mL NB on 499 mL deionized water)

Measure pH which should be around 6.8 - 7.2 and adjust, if necessary, with NaOH or HCL and sterilize by autoclaving. Store in the fridge at 5°C-10°C and use within one month after preparation.

Note

Variability introduced by complex media components

The variability shall be within the ranges given by ISO22196:2011. The meat extract and peptone used for the 1/500 NB test inoculum are the key components in minimizing variability. The following are the total nitrogen and α-amino nitrogen composition requirements for any commercially available material used for this International Standard. The peptone shall be an enzymatic digest of casein.

Meat extract: total nitrogen 6 % to 15 %; α-amino nitrogen 2 % to 5 %.

Peptone (enzymatic digest of casein): total nitrogen 12 % to 16 %; α-amino nitrogen 3 % to 6 %.

Plate count agar

**Compounds**	**CAS**	**1 L**	**PO[1]**	**Supplier**
Yeast extract	8013-01-2	2.5 g	2363	Roth
Tryptone	91079-40-2	5 g	48647-1KG	Serva
Glucose	14431-43-7	1 g	Merck	49159
Agar	9002-18-0	15 g	A0949	AppliChem

[1] the listed products are example products that were used to acquire the reference dataset

Adjust the pH of the plate count medium to 7.0 - 7.2 with NaOH or HCL and sterilize by autoclaving. Store in the fridge at 5°C-10°C and use within one month after preparation. A premix of the plate count agar can be purchased at Chemsolute (PO 9310-10X200ML).

2.3.2. Solutions

70% Ethanol

**Compounds**	**CAS**	**1 L**	**PO[1]**	**Supplier**
Ethanol	64-17-5	700 mL	2211-5L	Chemsolute

[1] the listed product is an example product that was used to acquire the reference dataset

Add 300 mL deionized water and mix.

Phosphate buffered stock solution

Compounds	CAS	1 L	PO[1]	Supplier
Potassium dihydrogen phosphate	7778-77-0	34 g	1648	Chemsolute

[1] the listed product is an example product that was used to acquire the reference dataset

Add 500 mL deionized water and mix to dissolve. Adjust the pH to 6.8 - 7.2 with NaOH and add deionized water to make up to 1000 mL. Sterilize by autoclaving and use within one month after preparation.

Phosphate buffered saline (PBS)

Prepare 10-fold physiological saline:

Compounds	CAS	1 L	PO[1]	Supplier
Sodium chloride	7647-14-5	85 g	1367	Chemsolute

[1] the listed product is an example product that was used to acquire the reference dataset

Add 1000 mL deionized water and mix to dissolve and sterilize by autoclaving. Dilute the phosphate-buffered stock solution to an 800-fold volume using the 10-fold physiological saline and water under sterile conditions. (e.g. 1.25 mL phosphate buffered stock solution mixed with 100 mL 10-fold physiological saline filled up with deionized water to 1000 mL). Store in the fridge at 5°C-10°C and use within one month after preparation.

Glycerol

Compounds	CAS	1 L	PO[1]	Supplier
Glycerol	56-81-5	600 mL	2039-1L	Chemsolute

[1] the listed product is an example product that was used to acquire the reference dataset

Add 400 mL deionized water, mix and sterilize by autoclaving.

Labdet 100

Compounds	CAS	1 L	PO[1]	Supplier
Labdet	-	50 mL	MED00003	Meintrup DWS

[1] the listed product is an example product that was used to acquire the reference dataset

Add 950 mL deionized water and mix.

Neutralizing solution

A valid neutralizer needs to be determined for different test coupons. Neutralization of the antibacterial activity of the copper test coupon can be achieved by immersing the test coupon into 10 mL NB medium.

2.4. Apparatus

Balance, capable of weighing to +/- 0.01 g

pH-meter, capable of measuring to +/- 0.2 units

Autoclave, capable of maintaining a temperature of 121 +/- 2°C and a pressure of 103 +/-5 kPA

100-1000 mL volumetric flask, suitable for autoclaving culture media and other solutions

Petri dishes with vents, sterile, 90 mm to 100 mm in diameter

Petri dishes without vents, sterile, 90 mm to 100 mm in diameter, suitable for withstanding hot culture medium

Square petri dishes without vents, sterile, with 120 mm x 120 mm dimensions, suitable for withstanding hot culture medium

Incubator, capable of shaking at 220 rpm and maintaining the set temperature of 35°C

Stoppered Erlenmeyer flasks (e.g. 100 mL) or media bottles for bacterial cultures

Fridge, capable of maintaining a temperature of < 8° C

Freezer, capable of maintaining freezer stocks of cultures at a temperature around -80 °C

Cryotubes (1.5 mL), suitable for the storage of bacterial cultures at -80 °C

Pipettes, capable of pipetting volumes between 10 µL and 1000 µL

Cover film, 40 x 40 mm hand/machine cut pieces from Stomacher bags or other films that does not affect bacterial growth or absorbs water, made of polyethylene, polypropylene or polyester [poly(ethylene terephthalate)]. A film that is 0.05 mm to 0.10 mm thick is recommended.

Stomacher bag or other plastic bags for neutralizing the inoculated sample in NB medium, rigid enough to withstand rupture.

Suitable rack, e.g. waste rack, capable of holding the stomacher bag during neutralization/sampling.

Wet chamber/Test chamber

> Gauze or absorbent cotton
>
> Metal grid, with dimensions of at least 300 mm x 200 mm to provide enough space for 6 test coupons in petri dishes with vents
>
> Sealable plastic boxes used as incubation chamber, with dimensions of at least 350 mm x 250 mm

Photometer, capable of measuring the optical density at OD_{600} of bacterial cultures.

Thoma counting chamber, capable of counting bacterial cells.

Microscope, capable of a resolution for counting of bacterial cells (400x magnification).

Test tubes, either 1.5 mL or 2 mL microcentrifuge tubes.

Vortex mixer, suitable for mixing test tubes.

Multichannel pipettes, capable of pipetting volumes between 10 µL and 200 µL.

96-well plate, made of polystyrene or polypropylene, capable of holding a volume of at least 200µL.

96-well plate shaker, capable of shaking 96-well plates for better mixture of the dilutions (can also be achieved by thoroughly resuspending with the multichannel pipette).

Glassware should be washed well with alkali or neutral detergent, then rinsed well with distilled or deionized water. All materials that come in contact with the culture or culture media should be sterilized by autoclaving prior to use.

3. Procedure

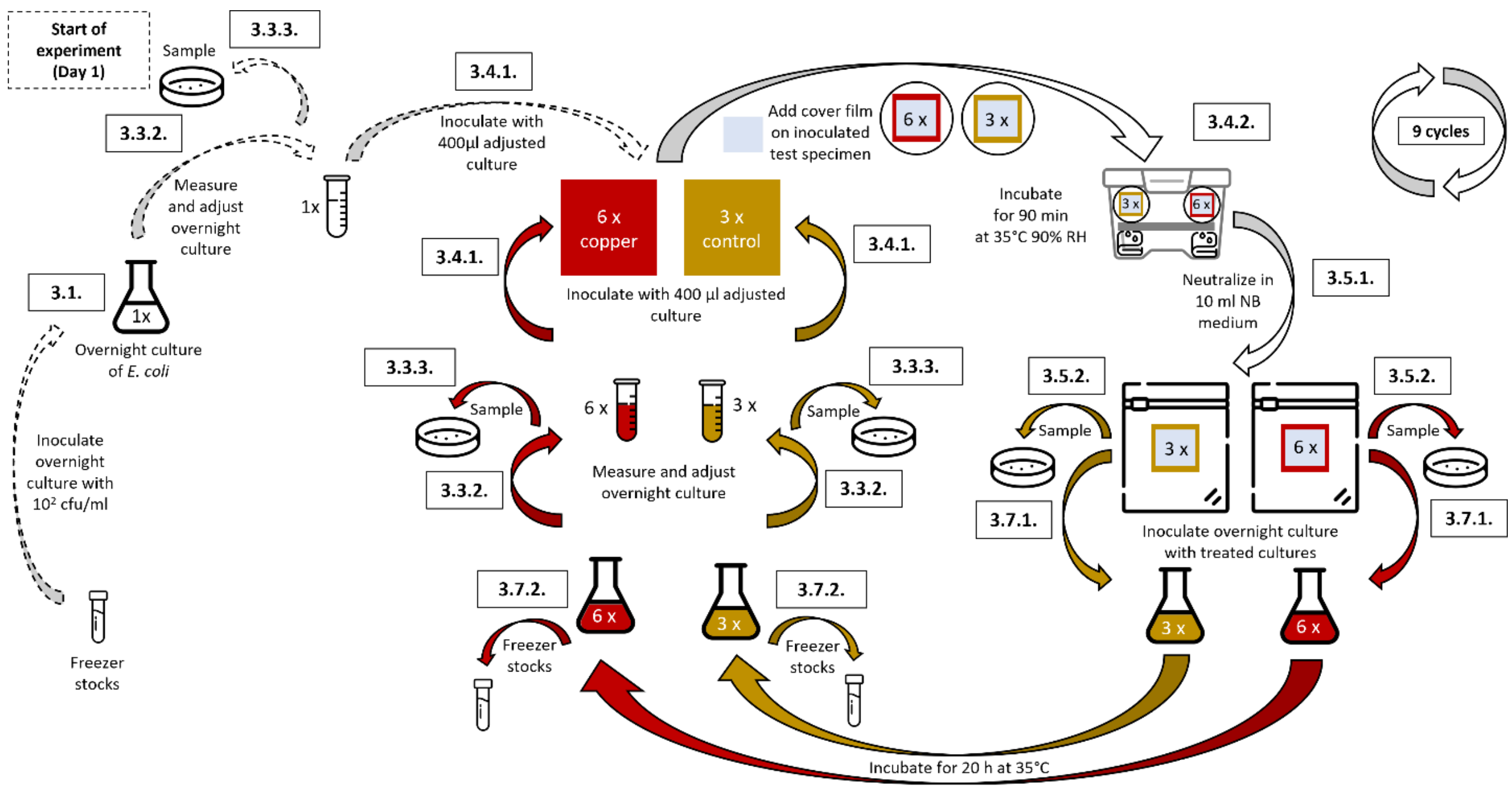

Figure S1. Scheme of the procedure of the resistance test method. Evolution will start from a single culture that will be split into 6 treatments exposed to the antibacterial surfaces and 3 control treatments exposed to the untreated surfaces. From this point onwards, the different lineages will be treated separately and any intermixing/cross-contamination must be strictly avoided. The figure contains elements (icons) used from flaticon.com.

3.1. Preparing bacterial stock culture

E. coli DSM1576 (ATCC8739) received from the culture collection should be transferred into 10 mL NB media and incubated overnight at 35°C with orbital shaking > 150 rpm. Measure the OD_{600} of the overnight culture, which should be around 2-6. The OD-cfu correlation (cfu = colony forming units) can be used to adjust the cell number by measuring the OD_{600} (3.3.2.). The expected OD-cfu correlation is for *E. coli* grown to stationary growth phase is 3 to 5 × 10^8 cells/mL at OD_{600} = 1.

Aliquot bacterial culture at a ratio of three parts bacterial culture and one part of 60 % glycerol in several cryotubes (0.75 mL bacterial culture and 0.25 mL 60 % glycerol solution). Repeated freeze-thaw cycles of aliquots should be avoided. Each aliquot used for inoculating an overnight culture should not be thawed more than 3 times.

3.2. Preparing the test coupons

3.2.1. Sterilization of the test coupons

Sterilize the test coupons (antibacterial and untreated) and cover films by incubating in 70 % ethanol for 15 min and afterwards let the coupons dry under sterile conditions (clean bench). Use for the test coupons separate container to avoid cross-contamination of the untreated coupons with antibacterial substances

leached from the antibacterial material. Transfer the sterile test coupons into sterile petri dishes with vents.

3.2.2. Setting up the test chamber and pre-warming of the test coupons

Set up a test chamber with the metal grid and wet cotton at 35°C and place the sterile petri dishes with vents with sterile test coupons into the wet chamber at least 1 h before starting the experiment in order to pre-warm the materials.

3.2.3. Cleaning of used test coupons after incubation

Disinfect the previously inoculated test coupons by incubating the antibacterial test coupons in 70 % ethanol for 15 min and the untreated test coupons in LabDet for 24 h. Use for the test coupons separate containers to avoid cross-contamination of the untreated coupons with antibacterial substances leached from the antibacterial coupons. Rinse with deionized water while rubbing the test coupon thoroughly by hand and dry the used materials with paper tissues.

3.3. Preparing the test inoculum

3.3.1. Cultivation of bacteria

Inoculate an overnight culture with 10^2 cfu from a freezer stock (see 3.1) in 10 mL NB medium in a 50 mL Erlenmeyer flask and incubate at 35 ± 1 °C with orbital shaking > 150 rpm for 16 h to 24 h. A cfu of 10^2 in the inoculated culture can be reached by preparing a 10^{-5} dilution of the bacterial stock culture (3.1.) and adding 100 µL of this dilution to 10 mL NB medium. The overnight culture should reach a final OD of at least 2 for further processing under 3.6. or 3.8. (tests before and after evolution) or an OD of 0.1 for further processing under 3.7. (evolution experiment).

3.3.2. Culture adjustment

Adjust the cell concentration of the culture to 2×10^8 cfu/mL (range 0.2×10^8 to 6×10^8 cfu/mL) in 1/500 NB **pre-warmed to 35°C**. This can be achieved by two approaches:

1. Cell counting with Thoma chamber at the microscope

Prepare a 1:100 and 1:1000 dilution of the overnight culture (3.3.1) in 1/500 NB medium.

Add 50 µL formaldehyde (36-38 % stabilized with methanol) to 950 µL cell suspension.

Count at least 5 squares of the appropriate dilution and calculate the cell concentration (cells/mL).

2. OD_{600} measurement with a photometer

The desired cell concentration of 2×10^8 cfu/mL approximately corresponds to $OD_{600} = 0.5$ (see 3.1.).

Dilute the overnight culture appropriately or at least 1:10 in 1/500 NB medium and measure the OD_{600} of the diluted cell suspension against 1/500 NB solution as blank.

Adjust the overnight culture to the specific cell number in 1/500 NB.

Use the adjusted culture immediately for inoculation on test coupons.

3.3.3. Determining the viable cell number of the adjusted culture

Determination of the viable cell number can be achieved by any microbiological standard methods suitable to obtain the cfu of a bacterial culture. It is advised to use the following method based on spotting: Sample 100 µL as 3 technical replicates from each adjusted culture into a 96-well plate. Prepare serial 10-fold dilutions for each sample down to 10^{-6} by diluting 20 µL culture in 180 µL PBS using a multi-channel pipette. Spot 10 µL of all dilutions on a square petri dish filled with plate count agar. Incubate the plate count agar plates at 35°C for 18 h. Count the colonies for those dilutions for which spots show a colony count of 3 to 50 cfu and calculate the cfu/mL using equation 1.

3.4. Inoculation of bacteria and incubation with the test coupons

3.4.1. Inoculation of bacteria

Transfer each sterile test coupon (antibacterial test coupons and untreated test coupons) into a sterile petri dish with vents. Add 400 µL of the adjusted culture with a 1000 µL pipette on the surface of the test coupon. Cover the inoculum on the test coupon with a cover film using tweezers and carefully spread inoculum evenly by pressing the film with the tweezers over the test coupon surface. Avoid leakage of the inoculum beyond the edges of the film. The cover film shall be 4 cm × 4 cm when covering test coupons that have dimensions of 5 cm × 5 cm.

3.4.2. Incubation of bacteria with test coupons

Close petri dish with vents and put into pre-warmed (see 3.2.2) wet chamber. Incubate petri dishes with vents with inoculated test coupons for the desired incubation time at 35°C in a wet chamber stuffed with wet cotton or wet paper tissues to maintain a humidity of 90%.

3.5. Sampling of bacteria from test coupons and determination of viable cell count

3.5.1. Removing bacteria from test coupons

Put a Stomacher bag over a suitable rack and fill it with 10 mL neutralizer (see 2.3.3 neutralizing solution). Add the incubated test coupons into the bag. Remove the cover film from the test coupon and rub the medium thoroughly all over the test coupon. If necessary, a pipette tip can be used to push the film from the test coupon. Be careful with sharp edges of test coupons because they can damage the Stomacher bag.

3.5.2. Determination of viable cell number

Determination of the viable cell number can be achieved by any microbiological standard methods suitable to obtain the cfu of a bacterial culture.

It is advised to use the following method based on plating or spotting:

Sample 200 µL as 3 technical replicates from each Stomacher bag into a 96-well plate after incubation of the cell suspension on the test coupon. Prepare serial 10-fold dilutions for each sample down to 10^{-6} by diluting 20 µL culture in 180 µL PBS using the multi-channel pipette. Spot 10 µL of all dilutions on a square petri dish filled with plate count agar. The target colony count is 3 to 50 cfu. In addition, plate 100 µL of the undiluted sample from the antibacterial test coupons on a round petri dish without vents filled with plate count agar. The target colony count is 1 to 300 cfu. Incubate the plate count agar plates at 35°C for 18 h. Count the colonies for those dilutions for which spots show a colony count of 3 to 50 cfu and count the plated undiluted sample if there is a colony count between 1 and 300 cfu. Calculate the cfu/mL using equation 1.

3.6. Determining the exposure time for the evolution experiment ('condition testing')

The appropriate exposure time of the test strain on the antibacterial test coupon should be determined prior to the evolution experiment (3.7). This is done by performing a time-kill experiment. This experiment is performed according to ISO22196:2011 with three major deviations: (i) use of an increased inoculum 0.2×10^8 to 2×10^8 cells/mL, (ii) inoculation of the pre-culture from a cell-suspension (not a colony) according to 3.3.1, and (iii) use of different incubation times on the antibacterial test coupon. To this end, perform steps 3.1 to 3.5 for different incubation durations including 24 h for 3 replicate antibacterial test coupons for each time point. The appropriate incubation time should result in R_i (initial antibacterial activity) of a minimum of 1.5 (± 0.5) or a maximum of 3.5 (± 0.5) (see section 4.3).

3.7. Evolution (ALEE-AMC)

3.7.1. Transfers and culturing for evolution

Perform steps 3.1 to 3.5.1. by preparing an inoculum from a single overnight culture (3.3.). Expose this inoculum to 6 replicate antibacterial test coupons and 3 replicate untreated test coupons. Transfer each test coupon into a separate Stomacher bag to remove the surviving bacteria (3.5.1.) and transfer 1 mL from each Stomacher bag into a separate Erlenmeyer flask pre-filled with 9 mL NB medium and incubate at 35 ± 1 °C for 16 h to 24 h.

Repeat from step 3.3.2. to 3.5.2. for 9 cycles for each of the 3 untreated lines and the 6 lines exposed to the antibacterial test coupons. It is advised to inoculate 3 test coupons at once and move them directly to the incubator (see below) to maintain an exact incubation time and to avoid disturbance of the incubation chamber.

The procedure allows to perform 3 cycles per working week. After the 3rd and the 6th cycle, the overnight cultures can be stored at 4°C for 3 days (i.e. from Friday to Monday). These cultures will be used to inoculate a fresh overnight culture on Monday and should be diluted by adding 100 µL to 10 mL fresh NB medium and incubating them at 35 ± 1 °C for 16 h to 24 h.

3.7.2. Sampling and storage of evolved cultures and isolation of evolved clones

Freezer stocks (see 3.1) should be prepared for each of the evolved lines after 9 cycles. Prepare 4 aliquots after the last cycle.

After the last cycle of the evolution experiment, the antibacterial activity along the experiment is evaluated and appropriate evolved populations with relevant differences in antibacterial activity are selected to perform 'adaption testing' (described under 3.8.). Relevant differences in antibacterial activity shall be assessed by the following condition: $R_{e1} \geq R_{e9} + 1$; comparing the mean reduction factor R_{e1} (similar to factor R_i) after the 1st exposure with R_{e9} after the last exposure calculated according to equation 4 (section 4.2).

If a relevant difference is apparent at the last cycle of the evolution experiment, then the liquid overnight cultures of two of the evolved lines with the highest reduction factor during the last exposure cycle (R_{e9}) to antibacterial test coupons shall be used to isolate one clone per line for 'adaptation testing' (described under 3.8.). If only one line with a relevant reduction factor is present, two clones shall be isolated from this line. In addition, two clones each from a different control lines exposed to untreated test coupons shall be isolated for 'adaptation testing' (described under 3.8.). If no relevant difference is apparent at the last cycle of the evolution experiment, then two clones from two non-adapted lineages shall be isolated.

To isolate the clones from the last treatment cycle, the liquid overnight cultures shall be appropriately diluted to expect single colonies and plated on plate count agar. From the resulting plates one or two single colonies shall be streaked on plate count agar and incubated overnight at 35°C. A single colony from each plate shall be used to inoculate an overnight culture incubated at 35°C with orbital shaking > 150

rpm. This overnight culture shall be used to inoculate a pre-culture for adaptation testing (3.8.) according to 3.3.1. In addition, the overnight culture is used to store a freezer stock of each clone as described in 3.1.

3.8. Determining adaptation of clones evolved in response to the test coupons (‘adaption testing’)

Adaptation of the clones isolated from the untreated control and the evolved lines (3.7.2.) shall be tested on the antibacterial material according to 3.6 at the incubation time determined under 3.6 and used under 3.7 by assessing the evolved antibacterial activity of a clone ($R_{e,ep1}$ and $R_{e,ep2}$ as well as $R_{e,cp1}$ and $R_{e,cp2}$, where the index *ep* denotes clones stemming from the evolved population and the index *cp* denotes clones stemming from the control population). To this end, perform steps 3.1 to 3.5 for 3 replicate antibacterial test coupons for all isolated, evolved clones (two of which evolved on the untreated and two of which evolved on the antibacterial test coupon).

4. Data Evaluation

4.1. Determination of the number of viable bacteria

The concentration of colony forming units (cfu/mL) is calculated according to equation 1:

$$F = \frac{C}{D \cdot V_S} \qquad \text{(Supplementary Equation 1)}$$

, where F is the concentration of cells after removing viable cells from the test coupon to the Stomacher bag (3.5.1.);

C are counts for the colony forming units (cfu) in individual spots or on individual plates;

D is the dilution factor for the spots or plates counted;

V_s is the volume, in mL, spotted (0.01 mL) or plated (0.1 mL).

Calculate F for each individual sample by selecting the countable spots or plates according to 3.5.2.

The variation between replicates of one treatment should follow the requirements for a valid test as described in ISO22196:2011 (section 8.2.2., equation 2)

For each incubated test coupon, determine the number of viable bacteria per test area recovered according to Equation (2):

$$N_r = \frac{F \times V_i}{A} \qquad \text{(Supplementary Equation 2)}$$

, where N_r is the number of viable bacteria recovered per cm^2 per test coupon;

V_i is the volume, in mL, of incubated cell suspension added to each test coupon (0.4 mL) plus the volume of neutralizer present in the Stomacher bag (10 mL);

A is the surface area, in cm^2, of the cover film (i.e. 16 cm^2 for coupons with 5 cm × 5 cm covered by cell suspension with 4 cm × 4 cm).

Calculate the geometric mean of the number of viable bacteria recovered for each set of test coupons from all 3 replicate treatments and express this value to two significant figures. If no colonies were recovered in any of the agar plates for a dilution series, then record the number of colonies counted as 'below detection limit'. When there are no viable bacteria recovered in a dilution series, use the calculated detection limit for the calculation of N (N=Vi/(Vs×D)).

The predicted number of viable bacteria initially present on the test coupon (N_a) is calculated from the cell concentration determination of the adjusted culture (3.2.3) according to equation 3:

$$N_a = \frac{F \times V_a}{A} \quad \text{(Supplementary Equation 3)}$$

, where V_a is the volume, in mL, of the adjusted cell suspension that was added to each test coupon (0.4 mL) at the start of the incubation.

4.2. Calculation of the antibacterial activity during evolution

The antibacterial activity on a test coupon according to

$$R = U_t \; - A_t \quad \text{(Supplementary Equation 4)}$$

,where R is the antibacterial activity,

U_t is common logarithm of the number of viable bacteria (retrieved from N_r), in cells/cm^2, recovered from the untreated test coupons at the end of the incubation time and averaged over all untreated test coupons;

A_t is common logarithm of the number of viable bacteria (retrieved from N_r), in cells/cm^2, recovered from the antibacterial test coupons at the end of the incubation time.

4.3. Calculation of the antibacterial activity before and after evolution

The initial (R_i) and evolved (R_e) antibacterial activity is calculated according to equation 4. R_i is the antibacterial activity at the beginning of the experiment and R_e is the antibacterial activity at the end of the experiment.

The experiments performed as described under 3.6. are used to calculate the initial antibacterial activity (R_i).

The experiments performed as described under 3.7. are used to calculate the evolved antibacterial activity of each replicate population and any given cycle number (R_{ex}), where x denotes the number of the cylce.

The experiments performed as described under 3.8. are used to calculate the evolved antibacterial activity of each different isolated clones ($R_{e,ep1}$; $R_{e,ep2}$; $R_{e,cp1}$; $R_{e,cp2}$), where the index *ep* denotes clones

stemming from the evolved population and the index *cp* denotes clones stemming from the control population.

4.4. Calculation of adaptation of the evolved treatments

The change in antibacterial activity due to adaptation (R_a) is calculated for each population at cycle 9 and each isolated clone according to equation 5 using the parameters described in section 4.3.

$$R_a = R_i - R_e \qquad \text{(Supplementary Equation 5)}$$
